# The Mediterranean Diet and Cardiovascular Protection: Biochemical Mechanisms with Emphasis on Platelet-Activating Factor

**DOI:** 10.3390/nu18091320

**Published:** 2026-04-22

**Authors:** Paraskevi Detopoulou, Smaragdi Antonopoulou, Pinelopi Douvogianni, Constantinos A. Demopoulos

**Affiliations:** 1 Department of Nutritional Science and Dietetics, University of Peloponnese, Antikalamos, 24100 Kalamata, Greece; p.detopoulou@uop.gr (P.D.); p.douvogianni@go.uop.gr (P.D.); 2 Laboratory of Biology, Biochemistry and Microbiology, Department of Nutrition and Dietetics, School of Health Science and Education, Harokopio University, 70 El. Venizelou Street, 17671 Athens, Greece; 3 Laboratory of Biochemistry, Faculty of Chemistry, National & Kapodistrian University of Athens, 16121 Athens, Greece

**Keywords:** mediterranean diet, cardiovascular diseases, biochemical mechanisms, Platelet-Activating Factor (PAF)

## Abstract

Landmark epidemiological studies and clinical trials, such as the Seven Countries Study, the Lyon Diet Heart Study, the PREDIMED Study and the CORDIOPREV Study, have shown significant reductions in cardiovascular events in those following the Mediterranean diet (MD). The aim of the present work is to summarize the most robust available evidence and the major biological pathways underlying the protective effects of the MD, with particular emphasis on the role of PAF inhibitors. Mechanistically, MD functions through a complex synergy of antioxidant, anti-inflammatory, and antithrombotic effects that collectively improve lipid profiles, enhance endothelial function, optimize postprandial metabolism and cell membrane signaling, making it a functional model for human longevity. The PAF-Implicated Atherosclerosis Theory has emerged as a key unifying framework, proposing that Platelet-Activating Factor (PAF)—a highly potent lipid inflammatory mediator—plays a central role in the initiation and progression of atherosclerosis. Oxidized LDL promotes the production of PAF and PAF-like lipids, leading to endothelial dysfunction, vascular inflammation, and atherosclerotic plaque formation. Traditional Mediterranean foods are rich in natural PAF inhibitors, particularly the polar lipid fractions of extra virgin olive oil, as well as wine, fish, vegetables, onions, and garlic. Animal studies demonstrate that these compounds can reduce or even regress atherosclerotic lesions, independently of serum cholesterol levels. Human dietary interventions have further shown that MD-based meals and functional foods enriched with PAF inhibitors reduce PAF activity and improve thrombosis-related biomarkers. This mechanistic framework helps explain phenomena such as the “French Paradox” and the cardio-protective effects associated with fish consumption. Moreover, the extraction of PAF inhibitors from Mediterranean food by-products, such as olive pomace, offers promising ecological and economic advantages. Collectively, targeting PAF and increasing dietary intake of PAF inhibitors represent promising strategies for the prevention and management of atherosclerosis and other inflammatory diseases, supporting the view that PAF may function as a major, modifiable risk factor in these conditions.

## 1. Introduction

According to the Global Burden of Disease, cardiovascular diseases (CVDs) are the primary contributor to both disability-adjusted life years and mortality [1]. Although age-adjusted rates of cardiovascular disease and mortality have been reduced in developed countries, the total burden continues to increase due to demographic expansion and aging [2]. The most common cause of cardiovascular disease is atherogenesis. Atherosclerotic plaque may cause obstruction of blood flow, resulting in ischemia, while, when it ruptures, it causes clots that can cause a heart attack or stroke. This article examines the protective role of the Mediterranean diet (MD) in cardiovascular diseases associated with atherogenesis and atherosclerosis, highlighting key intervention studies. In addition, it summarizes the most robust evidence available and the major biological pathways underlying this protective effect, with particular emphasis on the role of PAF inhibitors found in foods characteristic of the MD. The aim of this review is therefore to demonstrate that part of the cardio-protective effect of the MD may be attributed to the presence of micronutrients that act as PAF inhibitors, and to present evidence that the consumption of foods containing these compounds may represent an effective strategy for the prevention and management of atherosclerosis and other inflammatory diseases. Relevant studies were identified through non-systematic searches in PubMed and Scopus using terms related to MD, PAF and cardiovascular health.

It is estimated that almost 80% of CVD burden is attributable to modifiable risk factors, such as diet, smoking and exercise habits [1]. Within lifestyle modification, dietary habits and healthy dietary patterns, such as the MD, play a particularly important role in cardiovascular disease prevention [3].

## 2. Definition of the MD

### 2.1. The Multi-Level Definition of MD

The concept of the MD originated from the Seven Countries Study led by Professor Ancel Keys [4]. It was documented that dietary patterns and lifestyle practices developed over centuries among populations of the Mediterranean region were protective against CVD [4]. While regional variations exist, the diet’s foundation is rooted in the shared climatic and historical heritage of the Mediterranean basin, characterized primarily by the extensive use of olive oil for its anti-atherogenic properties [5]. MD also includes a high intake of seasonal vegetables, wild greens, fresh fruits, whole grains, and legumes, complemented by regular consumption of fish, nuts, and fermented dairy, while keeping red meats and processed foods to a minimum [6,7].

Modern clinical evidence now positions the MD as more than a simple nutritional plan. It encapsulates high nutritional, health, environmental, economic, social, and cultural value. This cultural framework encourages mindful eating and reduces stress [8]. In 2010, UNESCO recognized the MD as an Intangible Cultural Heritage of Humanity, honoring it as a vital cultural tradition [9]. Furthermore, the MD constitutes a holistic “way of life” that integrates healthy eating with physical activity and social connectivity to promote the health of people and the planet [10,11]. Indeed, the MD aligns with contemporary ecological priorities, in that, according to the World Wildlife Fund for Nature (WWF), the MD significantly reduces environmental footprint through sustainable consumption patterns [11]. Since it mostly relies on local, seasonal, and plant-based foods, it requires significantly less water, land, and energy compared to Western diets high in animal proteins. Economically, it supports local biodiversity and traditional farming practices. Moreover, it has earned recognition from the Food and Agricultural Organization (FAO) as a benchmark for sustainable nutrition [12]. Interestingly, sustainability is embedded in the newly designed Mediterranean pyramid, emphasizing local, seasonal, and minimally processed foods while discouraging food waste [13].

### 2.2. Assessment of MD Dimensions

Assessing adherence to the MD has evolved from simple food checklists to complex tools that capture the synergistic effects of the entire dietary pattern [14]. Researchers primarily utilize a priori scoring systems, where individuals receive points for consuming beneficial components (like olive oil, fruits, vegetables, whole-wheat products, legumes, etc.) and lose points for excess intake of non-traditional items (like red meat). The most commonly utilized scores are the Mediterranean Diet Score created by Prof Trichopoulou [15], the MedDietScore formulated by Prof Panagiotakos [16], and the MEDAS index developed for use in the PREDIMED study [17]. Some scoring in Spain and Italy also includes non-traditional foods such as sweets, processed foods and fast food as negative components in the scoring systems [14]. Beyond these predefined scores, exploratory data-driven pattern analysis allows researchers to identify a posteriori actual eating behaviors in a population, rather than comparing them to a theoretical ideal [18]. More recently, the field has moved toward “holistic” indices that integrate environmental sustainability and sociocultural factors, recognizing that the MD is a lifestyle. For example, the MEDLIFE index includes questions on food intake, Mediterranean dietary habits, physical activity, rest and social interaction [10].

## 3. The MD and Health

### 3.1. Official Recommendations on Health-Related Effects of the MD

The Italian Scientific Societies and the National Institute of Health Task Force on Clinical Practice Guidelines have launched 84 evidence-based recommendations regarding the beneficial effects of the MD [19]. More particularly, the MD was found to significantly reduce all-cause and cardiovascular mortality, and it was connected to a lower incidence of several types of cancer and improved outcomes. In addition, specific guidelines exist regarding the MD and its relation to the incidence and progression of cognitive decline, type 2 diabetes, metabolic syndrome, and obesity [19]. The European Guidelines on Cardiovascular Disease Prevention in Clinical Practice stress the importance of the adoption of the MD [3], while the MD is defined as a heart-healthy dietary pattern in the Scientific Statement from the American Heart Association [20]. The MD and the Dietary Approaches to Stop Hypertension (DASH) diet are also recommended for patients with hypertension to reduce their BP and CVD risk [21]. In parallel, the MD remains the preferred dietary pattern for the prevention and management of Type 2 diabetes due to its ability to improve glycemic control and heart health simultaneously, which is also stressed in the EU and US diabetes guidelines [22,23].

### 3.2. Landmark Studies Connecting the MD and CVD

#### 3.2.1. The Seven Countries Study

Launched in the late 1950s and spanning over five decades, the Seven Countries Study serves as an epidemiological landmark that examined 16 cohorts totaling 12,763 middle-aged men across seven countries (USA, Japan, Netherlands, Finland, Italy, Yugoslavia, and Greece). As the first major study to explore how cultural and dietary variations influence CVD, it revealed that populations in North America and Northern Europe experienced significantly higher rates of coronary heart disease compared to those in Japan and Southern European Mediterranean countries [4]. Both Greece and Japan exhibited low heart disease rates despite having vastly different total fat intakes, shifting the scientific focus toward fat quality and overall dietary patterns. After 25 years, the death rate from CHD was 8 to 10 times higher in Northern Europe and the US than in Japan and Mediterranean Southern Europe [24]. The study identified a high ratio of plant-based foods and fish relative to animal products and sugar as a primary protective factor. Evidence from the Seven Countries Study suggests that Cretan dietary patterns were a primary driver of their low disease rates [25], validating Ancel Keys’ famous advocacy for the “eat well and stay well” lifestyle [26]. Of note, the Laboratory of Food Chemistry of the National & Kapodistrian University of Athens participated under the leadership of the Director of the Laboratory, Professor D.S. Galanos and his collaborator Professor V.M. Kapoulas. The tradition of studying the relationship between the MD and olive oil (the main component of MD) with cardiovascular disease continued in the following years at the Laboratory of Food Chemistry.

#### 3.2.2. The Lyon Heart Study

The Lyon Diet Heart Study remains a cornerstone of nutritional epidemiology, serving as the first randomized controlled trial to demonstrate the efficacy of the MD in the secondary prevention of coronary heart disease. Following 605 patients who had survived a first myocardial infarction, the study compared a Mediterranean-style diet—supplemented with alpha-linolenic acid—with a standard prudent diet [27]. After 46 months of follow-up, 204 and 219 subjects participated in the control and intervention groups, respectively. Those following the MD had a 50% to 70% lower risk of recurrent heart disease, depending on the variables chosen to assess [27]. Due to the overwhelming statistical significance of the results, the trial was terminated prematurely for ethical reasons to allow the control group access to the dietary intervention [27].

#### 3.2.3. The PREDIMED Study

The PREDIMED (Prevención con Dieta Mediterránea) study is one of the most important randomized clinical trials in the field. It involved 7447 high-cardiovascular-risk participants with no disease at the start of the study. Participants were randomized into three groups [28]. The first group was assigned to an MD supplemented with extra-virgin olive oil (EVOO) (one liter of EVOO per week for the entire household). The second group followed the MD supplemented with mixed nuts (15 g walnuts, 7.5 g hazelnuts, and 7.5 g almonds) [28]. The third group served as the control, where participants were advised to follow a low-fat diet [28]. The PREDIMED Study showed that adopting the MD with the addition of EVOO or nuts leads to a significant reduction in the risk of cardiovascular events, approximately 30% compared to a low-fat diet [28].

#### 3.2.4. The CORDIOPREV Study

The CORDIOPREV study was a prospective, single-center, randomized controlled trial. The study enrolled 1002 patients with established coronary heart disease who followed an MD or a low-fat diet. The patients were followed for seven years, and it was documented that the MD was superior to the low-fat diet in preventing recurrence of major cardiovascular events (87 events in the MD group and 111 in the low-fat group). Multivariate models revealed that the MD consistently outperformed the low-fat intervention, with hazard ratios across various models ranging from 0.719 (95% CI: 0.541–0.957) to 0.753 (95% CI: 0.568–0.998) [29].

### 3.3. Meta-Analysis Connecting MD and CVD

Recent meta-analyses provide evidence that high adherence to the MD reduces CVD incidence in primary [30] and secondary prevention [31]. In particular, adherence to the MD significantly reduced CVD incidence (HR 0.76, 95% CI: 0.72–0.81) and coronary heart disease risk (HR 0.75, 95% CI: 0.65–0.87) in women [30] *(n* = 16 prospective studies). In secondary prevention (*n* = 19 studies), observational cohorts demonstrated a modest but statistically significant risk reduction (RR 0.95; 95% CI: 0.93–0.97) while randomized clinical trials (RCTs) revealed a far more profound effect, with a relative risk of 0.44 (95% CI: 0.20–0.94), suggesting that structured MD interventions may reduce recurrent events by up to 56% [31]. The long-term impact of MD on cardiovascular disease prevention is beneficial when both studies on primary and secondary prevention are considered [32]. A 2-point increase in MD adherence, assessed through a score, has been reported to reduce overall mortality by 8% and CVD risk by 10% [33]. Interestingly, in a network meta-analysis, the MD was the only dietary strategy capable of consistently reducing the risk of major cardiovascular events, myocardial infarction, angina, and all-cause mortality [34]. Similarly, according to umbrella meta-analysis, greater adherence to the MD reduced the risk of overall mortality, cardiovascular diseases, coronary heart disease, myocardial infarction and other diseases [35,36]. Meta-analyses of individual dietary components indicate that the cardio-protective benefits of the MD are primarily driven by the consumption of olive oil, fruits, vegetables, and legumes [37]. In a meta-analysis specifically focusing on the role of olive oil, it was found that for every additional 25 g/day of olive oil consumed, a 16% reduction in CVD risk is observed (RR 0.84; 95% CI: 0.76–0.94), underscoring a central role of this component in the diet–disease relationship of the MD [38].

## 4. PAF: From Chemical Elucidation to Its Pathogenetic Role

### 4.1. PAF Historical Aspects

**Figure 1:**
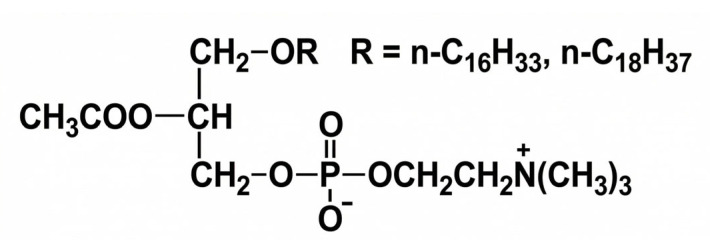
PAF structure.

In 1979, the PAF research group at the University of Texas at San Antonio elucidated the PAF structure (as 1-O-alkyl-2-acetyl-sn-glycero-3-phosphocholine) and subsequently synthesized it chemically (Figure 1). Named for its primary identified role in platelet aggregation, PAF has since emerged as the most potent lipid-based inflammatory mediator, exerting biological activity well beyond the scope of thrombosis [39].

PAF is the most potent lipid-based inflammatory mediator known today; it circulates in the body in very small concentrations, which made the elucidation of its structure as well its identification difficult. It is worth mentioning that, after its chemical synthesis by Demopoulos et al. [40], researchers were given the opportunity to have the required quantities of synthetic PAF to conduct experiments. This gave such an impetus for PAF research that a world conference was held every four years for twenty years, with the exclusive and sole topic of PAF. Research eventually showed that PAF is involved in almost all inflammatory diseases and many pathophysiological conditions. Upon the return of Prof. Demopoulos to Greece, this specialized knowledge was transferred, and the Greek PAF study group was established, comprising researchers from Greek universities, research centers, and state hospitals.

Today, PAF research is conducted globally. According to the Expertscape algorithm rankings for the last decade (2013–2023), approximately 2200 researchers in 600 academic/research institutions across 60 countries are involved in PAF research [41]. Among these countries, the USA ranks first, China ranks second, Japan ranks third, and Greece ranks fourth. Among institutions, the National & Kapodistrian University of Athens ranks first, Harokopio University of Athens ranks second, and Harvard University ranks third. Notably, of the top 10 positions among the 2200 researchers, seven are occupied by members of the Greek research team, holding the first, second, third, fifth, sixth, seventh, and ninth places, respectively [41].

### 4.2. The Biological Role of PAF

The real role of PAF in nature is to function as a lipid mediator (cellular signal) in animals, plants, and microorganisms [42]. Under homeostatic conditions, the spatio-temporal regulation of PAF is vital. For example, PAF is one of the first compounds produced during the fertilization of the female egg by the sperm [43]. However, PAF in the wrong amount and at the wrong time may be harmful to the organism (when it causes prolonged and undesirable inflammation for the organism) [39]. In other words, PAF levels are always under strict control thanks to regulatory mechanisms in its biosynthesis and degradation pathways. In addition, its circulating degradative enzyme, PAF acetylhydrolase, or Lp-PLA2, is a new risk factor for cardiovascular diseases; given its sensitivity to PAF levels, the design of its specific inhibitors has become a therapeutic target of interest [44]. From the first studies of PAF in experimental animals, it was found that intravenous administration of synthetic PAF to non-sensitized rabbits caused complete allergic shock with all the pathobiological effects, i.e., the same vascular–cardio–respiratory symptoms associated with anaphylaxis [45]. Since then, the involvement of PAF in many diseases, such as allergies, cancer, cardiovascular and kidney diseases, hepatitis, diabetes, HIV infection, COVID-19, damage to the central nervous system, etc., has been proven or suggested [39,46,47,48,49].

It is noted that, to date, although there are many natural and synthetic PAF inhibitors [50,51], only one drug is currently circulating with the active ingredient rupatadine, which is one such PAF inhibitor [52]. In addition, there are other drugs (e.g., anti-inflammatory, antiviral) that inhibit PAF among their pleiotropic actions [53,54,55].

## 5. Mechanistic Aspects of Atherosclerosis with an Emphasis on PAF

Before turning our attention to the specific biochemical mechanisms that offer an explanation for the beneficial effects of the MD in atherosclerosis, it is essential to revisit its core concepts and highlight the biological significance of PAF and related compounds in its development. A significant amount of atherogenesis theories center on the “response to injury” hypothesis by Russell Ross [56], which emphasized vascular endothelial disturbance as the initiating event that triggers inflammation, oxidized low-density lipoprotein (LDL) accumulation, and smooth muscle cell proliferation, resulting in plaque formation. During the past few decades, several theories were proposed to explain the initiation of the atherogenic process, with the theory of oxidative modification of LDL being particularly noteworthy. The oxidative modification theory, introduced by Brown and Goldstein [57], proposes that an imbalance between the production of reactive oxygen and nitrogen species (ROS/RNS) and antioxidant defense systems results in excessive ROS/RNS generation, promotes LDL oxidation, increases LDL atherogenicity by altering receptor-mediated uptake by cells in the intima of blood vessels, and thus, promotes atherogenesis initiation. Recent data propose that LDL infiltration into the subendothelial space is facilitated not only by receptor-mediated transport, such as LDL Receptor (LDLR) and Scavenger Receptor B1 (SR-B1), but also by caveolae-mediated transcytosis [58,59]. The oxidation of LDL (low-density lipoprotein, which is rich in cholesterol) is a multifactorial process involving modifiable factors such as diet (antioxidants and bioactive ingredients in food) and lifestyle factors (such as smoking, sedentary lifestyle, etc.). The oxidative modification of LDL produces PAF and oxidized lipids with PAF-like activity in an uncontrolled way [60], which was isolated and identified both chemically and biologically [61], and also significantly reduces the activity of the degradative enzyme, Lp-PLA2 (lipoprotein-associated phospholipase A2), which renders PAF into an inactive biological molecule [62]. In addition, the “mevalonate hypothesis” proposes that inflammatory stimuli trigger the mevalonate pathway within endothelial cells, leading to the production of cholesterol and reactive oxygen species. According to this theory, atherosclerosis is not caused by the cholesterol itself, but rather by the free radicals, which in turn facilitate the creation of oxidized cholesterol [63].

Current insights view various atherogenesis theories as complementary, not mutually exclusive, with a strong emphasis on atherosclerosis as a chronic inflammatory vascular disease initiated by endothelial dysfunction and driven by oxidized lipids and hemodynamic forces, involving complex immune responses and cellular changes. In unresolved inflammatory conditions, as well as disturbed flow-derived biomechanical stimulation, the endothelium undergoes a sustained phenotypic modulation, known as endothelium type II activation [64].

In addition, platelets play a pivotal role in linking inflammatory mechanisms to the development of atherosclerotic disease. Their contribution extends well beyond facilitating immune cell recruitment to the vascular wall [65]. Among the mediators released by activated platelets, as well as by the endothelial cells, PAF has emerged as a critical regulator of vascular inflammation. PAF functions as a potent signaling molecule that triggers endothelial dysfunction [66,67], and also strengthens adhesive interactions between leukocytes, platelets, and the endothelial surface, promoting their stable attachment and retention at sites of vascular activation [68]. Additionally, PAF rapidly decreases nitric oxide (NO) production in vascular endothelial cells, further contributing to endothelium dysfunction [69]. Augmented PAF levels during unresolved inflammatory conditions lead not only to the aforementioned short-term actions but also to long-term (angiogenesis) alterations of the endothelial cells [70]. Furthermore, injured endothelial cells synthesize PAF that orchestrates responses in immune-competent cells like mast cells, neutrophils, monocytes, and lymphocytes, acting as a crucial signaling molecule for inflammation [71,72].

Oxidative modification of LDL leads to the formation of oxidized phospholipids (OxPLs) that can interact with the PAF receptor (PAF-R). These OxPLs are capable of eliciting biological responses comparable to those induced by PAF and are therefore classified as molecules with PAF-like activity [73]. Moreover, accumulating evidence indicates that signaling triggered by the PAF–PAF-R complex can activate Toll-like receptor 4 (TLR4), with additional data suggesting a similar crosstalk with Toll-like receptor 2 (TLR2) [74]. Experimental and clinical studies further demonstrate that TLR4 and TLR2 expression are markedly increased in atherosclerotic lesions across multiple vascular cell types. In this pathological setting, activation of these receptors appears to be driven predominantly by endogenous ligands, such as PAF and OxPLs, rather than by exogenous microbial stimuli [75].

Lp(a) constitutes another atherogenic lipoprotein carrying the largest fraction of oxidized phospholipids [76]. Interestingly, in patients with CVD, Lp(a)-associated PAF-AH (PAF’s catabolic enzyme) exhibits significantly lower mass, specific activity, and kinetic efficiency compared to controls or hypercholesterolemic patients [77]. This may impair the ability of Lp(a) to degrade pro-inflammatory phospholipids and may contribute to the acceleration of CVD [77].

Collectively, these findings support and reinforce the concept of a central role for PAF in atherosclerosis, as originally proposed in “The PAF-Implicated Atherosclerosis Theory” (2003). This seminal work, featured on the cover of Journal of Lipid Science and Technology (Vol. 105, Issue 11), identifies PAF as a primary etiological factor in atherogenesis [61]. The theory integrates previously proposed mechanisms of atherosclerotic disease (inflammation, thrombosis and oxidation), which can, however, be unified through the actions of PAF and highlights the inflammatory mediator PAF as a unifying and pivotal molecule in the initiation and progression of atherosclerosis. The “PAF-mediated Atherosclerosis Theory” has also been animated in a YouTube video (https://youtu.be/yKrWBA7l7s4, accessed on 19 April 2026).

## 6. How the MD Protects Against Atherosclerosis: Potential Mechanisms

**Figure 2:**
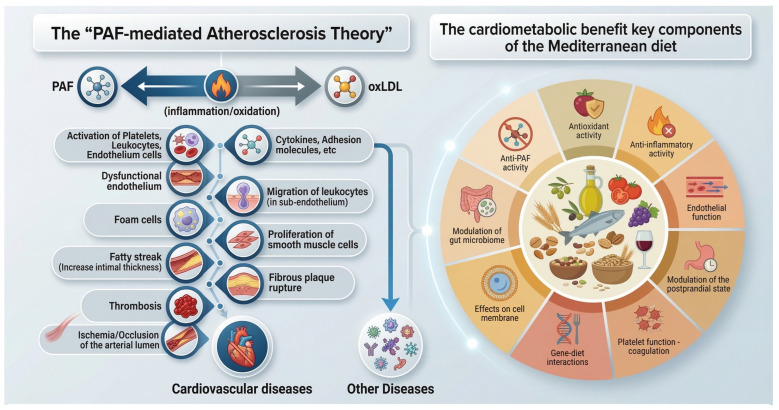
Potential mechanisms through which MD protects against atherosclerosis. An oversimplified account of the involvement of PAF in atherogenesis and the resulting cardiovascular diseases, though not limited to these diseases, is shown. The role of PAF in CVD is described in detail in the YouTube video, https://youtu.be/yKrWBA7l7s4, accessed on 19 April 2026.

A plethora of research data suggests that the MD, a plant-based diet with EVOO, wine and fish, leads to a reduction in the incidence of cardiovascular diseases caused by atherosclerosis and increased life expectancy. In addition, the synergistic consumption of these foods seems to impact metabolic pathways, helping alleviate the incidence of disease, morbidity, and mortality. Although MD has been a subject of study for many decades across various scientific fields, the biochemical mechanism through which it exerts its beneficial effects on different diseases has yet to be fully clarified. Mechanistically, the diet functions through a complex synergy of antioxidant, anti-inflammatory, and antithrombotic effects that collectively improve lipid profiles, enhance endothelial function, optimize postprandial metabolism and cell membrane integrity, making it a definitive model for human longevity. In addition, the MD may affect responses in persons with specific SNPs or genetic scores (Figure 2).

### 6.1. Antioxidant Activity

The MD is naturally high in antioxidant compounds, particularly phenolic compounds (phenols and polyphenols, such as hydroxytyrosol, oleuropein, resveratrol, and quercetin), vitamins (e.g., C and E), carotenoids, and other phytochemicals abundant in EVOO, fruits, vegetables, nuts, whole grains, and red wine. These compounds can directly scavenge reactive oxygen species (ROS), thereby decreasing oxidative damage to lipids, proteins, and DNA. The enrichment of LDL with phenolic compounds decreases its susceptibility to oxidation and aggregation [78]. For example, the European Food Safety Authority (EFSA) acknowledges that a daily intake of 5 mg of hydroxytyrosol and its derivatives is sufficient to protect LDL particles from oxidative damage [79]. Antioxidants also prevent the formation of oxLDL containing PAF and oxidized lipids with PAF-like activity [62] (see Section 8.2 for more details). In addition, EVOO and red wine phenolic compounds stimulate the transcription factor nuclear factor erythroid-derived 2-related factor 2 (Nrf2), which increases the expression of antioxidant and phase-II detoxification enzymes, enhances glutathione metabolism, and reduces ROS generation [80]. Human pharmacokinetic data suggest that nutritional doses (approximately 250–500 mg/day in supplemental form, or lower via dietary wine) can achieve peak plasma concentrations in the nanomolar to low micromolar range up to 2 μM [81], which is sufficient to trigger the Nrf2-mediated antioxidant response [82]. MD foods rich in these micronutrients contribute to a higher overall antioxidant capacity and higher levels of antioxidant vitamins such as ascorbic acid, tocopherols, and carotenoids compared with typical Western diets [83]. Of note, phenolic compounds also reduce cooking-related oxidants. For example, the addition of herbs, vinegar and olive oil has been shown to increase the dish’s antioxidant capacity [84] while EVOO is less prone to oxidation due to its monounsaturated fatty acid content [85]. The PREDIMED study showed that both MD groups exhibited a shift toward a more antioxidant profile compared to the low-fat diet group, as measured by plasma total antioxidant activity as well as by reduced oxidized LDL and malondialdehyde (MDA) levels in mononuclear cells [86,87].

### 6.2. Anti-Inflammatory Activity

A recent umbrella review of systematic reviews and meta-analyses showed that the MD reduces the levels of inflammatory markers C-reactive protein (CRP) (reduction of −0.37 to −1.04 mg/L), interleukin-6 (reduction of −0.38 to −1.07 pg/mL), and increases adiponectin (increases of 0.59 to 1.69 µg/mL) [35]. It is well known that PAF induces the production of cytokines (IL, etc.), which in turn can feed back and promote PAF production [39]. Of note, low-fat diets, as well as vegetarian and vegan dietary patterns, have produced inconsistent results, showing variable associations with CRP and no consistent effects on IL-6 or other proinflammatory markers, indicating the unique lipid composition of the MD in its anti-inflammatory action [35,88]. Table 1 presents the results of a meta-analysis of RCTs in high-risk individuals (obesity, CVD, and NAFLD) regarding the effects of the MD on inflammatory markers. In most RCTs, the MD was compared to a low-fat or habitual diet.

This anti-inflammatory action stems in part from phenols in EVOO and polyphenols in fruits, vegetables, and wine. Several compounds suppress pro-inflammatory signaling pathways (e.g., the pro-inflammatory transcription factor nuclear factor κB, NF-κB), improve metabolic health by upregulating AMPK pathways, and deregulate nicotinamide adenine dinucleotide phosphate (NADPH) oxidase-mediated oxidative stress [92]. Studies in cell cultures have shown that phenolic compounds derived from EVOO and red wine suppress inflammatory angiogenesis in endothelial cells by inhibiting the activity of Matrix Metalloproteinase-9 (MMP-9) and Cyclooxygenase-2 (COX-2), highlighting their potential to protect against atherosclerotic vascular disease [93]. In a double-blind randomized crossover trial of 24 young women with high-normal blood pressure, daily consumption of 60 mL polyphenol-rich EVOO (564 mg/kg polyphenols, providing ~30 mg/day) for 2 months significantly reduced C-reactive protein by 1.9 mg/L [94]. Olive oil polar lipids, which function as PAF antagonists, work alongside α-tocopherol and particular phenols to suppress PAF-mediated pathways, thereby contributing to the anti-atherogenic potential of olive oil [5]. Omega-3 fatty acids from fish further contribute by modulating lipid metabolism, reducing triglycerides and blood pressure and attenuating inflammatory cytokine production [95]. More particularly, they lead to the production of anti-inflammatory lipid mediators, i.e., resolvins, protectins and maresins [96]. In addition, omega-3s favorably affect the phospholipid composition of the cell membrane, and they inhibit the activation of NF-κB, thereby reducing the expression of inflammatory genes [96]. Moreover, they activate the anti-inflammatory transcription factor peroxisome proliferator-activated receptor γ [96] and they reduce PAF production [97]. Additionally, the MD’s high fiber content alters the gut microbiome and increases production of short-chain fatty acids (SCFAs) that mediate systemic inflammation and metabolic regulation [98]. The MD effects on gut microbiomes are further explained in Section 6.8. The effects of MD components on PAF actions and metabolism will also be separately analyzed in Section 8 (The role of diet in modulating PAF synthesis and activity: a unifying mechanistic approach against CVD).

### 6.3. Endothelial Function

The MD and its components may be protective by improving NO bioavailability and reducing inflammation and oxidative stress [91]. Green leafy vegetables and other foods provide a secondary source of NO via the nitrate–nitrite pathway. This mechanism compensates for NO deficiencies that occur when the body’s internal NO synthase system dysfunctions, thereby preserving essential NO levels [99]. Olive oil phenolic extract, oleuropein aglycone, and hydroxytyrosol reduce ICAM-1 and VCAM-1 cell surface expression at IC50 < 1 μM, which represents physiologically relevant concentrations [100]. Indeed, the MD has been shown to improve endothelial function in high-risk individuals [101,102] and individuals with cardiovascular disease [103], while no effect was reported in healthy individuals [104]. Moreover, the MD may positively influence endothelial regenerative capacity, as reflected by the release of endothelial microparticles [105]. A meta-analysis showed that the MD improved flow-mediated dilation (FMD) by 1.66% [106]. Notably, a synergistic effect on the improvement of FMD has also been observed following the combined intake of red wine and green olive oil in healthy young men [107]. MD has also been reported to reduce the levels of circulating adhesion molecules, indicative of endothelial activation and dysfunction, such as intracellular adhesion molecule 1 (ICAM-1) [90,108]. A recent meta-analysis has also shown beneficial effects of the MD on P-selectin and vascular cell adhesion molecule 1 (VCAM-1) [90].

### 6.4. Modulation of the Postprandial State

Impaired postprandial metabolism, characterized by significant fluctuations in blood glucose and lipid levels, is a recognized precursor to low-grade inflammation [109] and cardiovascular disorders [110]. The adherence to the MD and the MD lifestyle effectively blunts these post-meal disturbances by lowering circulating glucose, insulin, triacylglycerols (TAGs), and apolipoprotein B-48 levels [111,112,113,114]. The high fat and fiber content of the MD, as well as several traditional add-ons like cinnamon and vinegar, reduce gastric emptying, leading to a lower glycemic response [115,116,117]. The accumulation of advanced glycation end products (AGEs) resulting from glycoxidative stress is also a significant factor in the development of chronic diseases, while their restriction has beneficial effects [118]. Evidence suggests that adherence to an MD can modulate the production and accumulation of AGEs during cooking [119]. The addition of olive oil in cooking may enhance the bioactive lipid content of ingested foods, while cooking methods and increased temperatures may induce detrimental changes in the fish lipid profile, which may reduce its cardio-protective effects [120]. In addition, postprandial studies show that EVOO containing 1125 mg polyphenols/kg and 350 mg tocopherols/kg significantly lowers postprandial net incremental area under the curve for sICAM-1 and sVCAM-1 in both healthy subjects and subjects with dyslipidemia [121].

Research indicates that MUFAs help control postprandial blood lipid levels [122], while omega-3 fatty acids reduce post-meal triacylglycerols, possibly through enhanced chylomicron clearance and reduced VLDL secretion [116,123]. Data from our research team showed that the consumption of wild greens of Crete on top of a meal with bread and olive oil lowered postprandial glucose levels in healthy subjects [124]. Moreover, emerging evidence suggests that bioactive microconstituents present in wine—that also act as PAF inhibitors—may attenuate the hypertriglyceridemic effects of ethanol. Specifically, studies have shown that wine consumption results in a smaller increase in TAG concentrations compared with an equivalent ethanol solution, and that gin—but not red wine—significantly elevates the TAG area under the curve (AUC) relative to water [125,126].

Clinical and mechanistic studies indicate that the MD reduces postprandial coagulation responses more effectively than low-fat diets enriched with omega-3 polyunsaturated fatty acids, with additional synergistic effects derived from nitrate-rich vegetables and moderate red wine consumption. Indeed, postprandial physiology typically involves a significant rise in Factor VII coagulant activity approximately 2 to 3 h following the ingestion of a high-fat meal, which is attenuated in the chronic consumption of a MUFA-rich diet [99]. In addition, a 90-day MD intervention has been associated with lower increases in postprandial hemostatic factors (fibrinogen, factor VIIc, and factor VIIIc) [127]. Postprandial platelet aggregation may also be acutely affected, as evidenced by the consumption of wild greens with a high-fat meal in patients with metabolic syndrome [128]. In healthy male participants, red wine intake was associated with a significant attenuation of platelet aggregation induced by PAF 5–6 h post-consumption, compared with both an alcohol-matched ethanol solution and water, suggesting that this effect is attributable to non-ethanolic microconstituents of wine [125].

### 6.5. Platelet Function—Coagulation

MD adherence is related to lower levels of coagulation markers, such as D-dimer, homocysteine, and fibrinogen [129,130,131]. An MD for 12 weeks led to decreases in the antigen levels of coagulation factor VII and factor VIII [132]. Interestingly, supplementation of a saturated fatty acid-enriched diet with 15% olive oil (a higher amount than naturally occurring in the human diet) reduced vascular thrombogenicity and platelet activation in rabbits [133]. On the contrary, saturated fats have been linked to increased platelet aggregation [134]. In a subsample of the PREDIMED Study, an MD improved atherothrombosis biomarkers in subjects with high CVD risk, evidenced by reductions in fibrinogen and increases in PAF acetylhydrolase in high-density lipoproteins, which is the enzyme that catabolizes PAF [135]. In vitro studies support an anti-thrombotic effect of traditional MD foods and herbs (such as sweet basil) [136,137]. In an intervention study, the consumption of traditional Greek MD meals for ~1 month reduced platelet activity in healthy subjects and patients with type 2 diabetes [138]. Similarly, the consumption of a Mediterranean-type fast-food diet rich in PAF antagonists improved the platelet response of subjects with type 2 diabetes and healthy volunteers, as reflected by increases in the EC(50) values of PAF and other agonists of platelet aggregation [139]. The consumption of foods rich in PAF inhibitors (yogurt enriched with polar lipids from olive oil by-products (OOPLE) and gilthead sea bream fed with OOPLE-enriched feed) has also led to reductions in platelet aggregation stimulated by PAF and ADP and activated partial thromboplastin time [140,141]. This aspect will be separately analyzed in Section 8.

### 6.6. Gene–Diet Interactions

MD adherence has been linked to beneficial changes in DNA methylation patterns, particularly in genes related to inflammation and mitochondrial function [142]. Several nutrient–gene interactions have been reported. A higher MD adherence significantly modulates genetic susceptibility to weight gain. Individuals with a high Genetic Risk Score (GRS) for obesity showed a markedly lower risk of becoming obese when following an MD [143]. The effect of the MD in specific genetic SNPs, such as those of the FTO gene, is controversial [144,145,146,147]. The MD may also influence CVD by interacting with the MTHFR 677CT genotype, which helps normalize homocysteine concentrations, as evidenced by the ATTICA Study [148].

In addition, MD is now recognized as a potent regulator of circulating and exosomal microRNAs (miRNAs). These small, non-coding RNA molecules fine-tune the expression of genes involved in plaque formation, inflammation, and vascular repair. The CORDIOPREV trial suggests that baseline miRNA levels can predict how well a patient will respond to the MD. More particularly, the MD was particularly effective at reversing Type 2 diabetes for individuals with elevated baseline levels of miR-141-5p, miR-182, and miR-192 [149]. In addition, phenolic compounds found in EVOO can modulate the expression of specific microRNAs (miR-155-5p and miR-34a-5p) that regulate genes involved in adipocyte inflammation and vascular health [150]. The MD may also reduce cardiovascular risk by upregulating key genes involved in cholesterol transport, specifically NR1H2, PPARD, and ABC transporter. In this way, cholesterol efflux is facilitated, and cholesterol is removed from endothelial cells [151]. As evidence increases, in the future, it may be possible to use an MD approach as personalized nutrition guidance [152].

### 6.7. Effects on Cell Membrane

In a cross-sectional analysis of 1859 participants from the PREDIMED study and 6868 participants from the NHS/HPFS cohorts, a metabolic signature of MD adherence was identified, predominantly composed of lipid species and acylcarnitines (67%) [153]. Of note, specific subsets of these metabolites were consistently associated with higher intakes of olive oil, wine, and fish/seafood, lower consumption of sugar-sweetened beverages and sweets, and reduced CVD risk [153]. Importantly, the association between this metabolic signature and CVD risk persisted after exclusion of individual metabolites or unsaturated lipid species containing eicosapentaenoic acid (EPA), docosahexaenoic acid (DHA), or docosapentaenoic acid (DPA), indicating cumulative and synergistic effects across multiple MD components [153].

Concordant results were observed in the Reduction of Metabolic Syndrome in Navarra (RESMENA) trial, which enrolled 72 obese individuals with at least two metabolic syndrome features. This study evaluated plasma metabolomic changes following short- and long-term dietary interventions based on an energy-restricted MD or a low-fat diet. MD adherence was associated with marked alterations in circulating metabolites, particularly glycerophospholipids, including phosphatidylcholines (PCs) and lysophosphatidylcholines (LysoPCs) [154]. Further evidence from a subgroup of 772 hypertensive participants in the PREDIMED trial demonstrated that a one-year MD intervention reduced the cholesterol-to-phospholipid ratio and increased phosphatidylethanolamine content in erythrocyte membranes [155].

Collectively, these findings support a role of MD in remodeling membrane lipid composition and its biophysical properties, such as membrane fluidity, membrane protein function and receptor-mediated signaling [156]. In parallel, whether acting as a direct membrane building block [157,158] or indirectly by shifting the cellular inflammatory milieu and fatty acid production during metabolism [39], PAF plays a central role in maintaining or altering membrane integrity. In addition, membrane damage stimulates the production of PAF and various PAF-like agonists, all of which exert their biological effects by binding to the PAFR [159]. PAFR stimulation promotes the translocation of aSMase to the plasma membrane, where it catalyzes the hydrolysis of sphingomyelin. The resulting accumulation of ceramide—coupled with the subsequent generation of microvesicles—modifies the physical properties of the membrane, specifically its integrity and fluid dynamics [160,161,162]. Of note, PAF levels and the activity of its metabolic enzymes have been associated with red blood cell fatty acids [163]. In addition, PAF levels have been negatively associated with phase angle, a marker of cellular health, while the dietary antioxidant capacity of the diet was positively associated with phase angle [164].

### 6.8. Modulation of Gut Microbiome

The gastrointestinal microbiome functions as a dynamic metabolic system, mediating the effects of habitual dietary patterns on systemic inflammation, lipid metabolism, and pro-thrombotic pathways that contribute to the development of atherosclerosis [165,166,167,168]. Thus, the MD, characterized by abundant consumption of minimally processed plant-based and fermented foods, is considered a rich source of fermentable fibers and biologically active compounds (dietary fiber, phenolic compounds, and unsaturated lipids) that can influence microbial ecology and metabolic output [169,170,171,172]. Recent studies show that closely following the MD is often linked to a more diverse gut microbiome, with a higher presence of bacteria known to support health (many of which are SCFAs), and decreased representation of pro-inflammatory microbial signature types. However, the specific taxa and effect sizes vary across studies due to differences in populations, diet definitions, and microbiome methodologies [98,173,174]. In a similar vein, Mitsou et al. demonstrated that adherence to the MD was associated with distinct gut microbiota characteristics and variations in gastrointestinal symptoms [175].

The greater intake of dietary fiber and resistant starch present in the MD would result in the increased production of SCFAs produced by the gut microbiota (acetate, propionate, and butyrate), which have diverse biological functions that contribute to athero-protective processes [176,177]. SCFAs maintain gut barrier function, promote mucus secretion, and influence immune responses and cytokine production [178,179].

The gut microbiome produces additional metabolites implicated in atherosclerosis, such as trimethylamine N-oxide (TMAO). TMAO is formed through a sequential process in which gut microbes first metabolize nutrients such as choline, phosphatidylcholine, L-carnitine, and betaine into trimethylamine (TMA). This intermediate is oxidized in the liver and forms TMAO [180]. Multiple studies have linked higher circulating levels of TMAO to greater cardiometabolic risk, while choline intake has been inversely linked to inflammation [181]. TMAO drives cardiovascular disease by disrupting cholesterol transport, fueling macrophage foam cell formation, and promoting vascular inflammation and platelet hyperactivity [180,182,183,184,185]. These prothrombotic mechanisms are particularly relevant to an atherothrombotic phenotype, as both experimental and translational studies suggest that TMAO can increase the responsiveness of platelets and their potential for thrombosis, while recent reviews of the literature on the “gut–heart axis” describe converging pathways involving oxidative stress signaling and altered responses to antiplatelet therapies [185,186,187,188].

The MD is likely to decrease the risk of TMAO-related disease through both the lower intake of certain precursors (particularly those from red and processed meats) and by modulating gut microbiota composition and function [189,190,191,192]. Some MD-based interventions report decreases in TMAO or expect a lower burden of TMAO when consuming the MD, while others have observed little or no change in plasma TMAO despite adherence to the MD. Regardless, nested studies of MD trials show that choline pathway metabolites and TMAO-related signatures correlate with incident cardiometabolic events, highlighting the clinical significance of TMAO biology regardless of whether dietary responsiveness to the MD varies between cohorts [193]. Therefore, it seems most reasonable to conclude that the MD will tend to alter the overall gut metabolic environment to one that is less conducive to a pro-atherothrombotic state; although, the degree to which individual responses in TMAO will vary, based on the specific dietary pattern and host factors, cannot be fully predicted at this time.

Additional microbial mechanisms that may contribute to the cardio-protective effects of the MD include bile acid and tryptophan-derived signaling. Gut microbes convert primary bile acids into secondary forms, which interact with receptors such as FXR and TGR5. This interaction can influence lipid and glucose metabolism, while also affecting inflammation and the way the body processes lipoproteins [194,195,196]. Similarly, gut microbial metabolism of tryptophan results in the generation of indole derivatives, which may contribute to enhanced gut barrier integrity and regulation of immune responses through aryl hydrocarbon receptor signaling [197]. These axes are increasingly viewed as important contributors to diet–microbiome–host interactions that may impact the initiation of atherosclerosis and plaque biology [198].

Low-grade endotoxemia, characterized by the movement of bacterial lipopolysaccharides (LPSs) from the gut into circulation, has been observed in individuals at higher risk for cardiometabolic diseases. This condition has been linked to increased platelet activation, impaired endothelial function, and the progression of atherothrombotic events [199,200]. Mechanistically, LPS stimulates host cells (such as monocytes and endothelial cells) and stimulates them to produce a variety of lipid mediators, including PAF [201,202]. Therefore, there appears to be a possible pathway for how disruptions of the gut barrier lead to innate immune activation and enhanced vascular inflammation and thrombosis [203,204]. Additionally, microbial metabolites (such as TMAO) that enhance platelet hyper-reactivity may act in concert with PAF-dependent mechanisms—it has also been noted that the gut microbiome produces PAF inhibitors as well—at both the platelet and endothelium–leukocyte interface, and may therefore influence the degree of thrombo-inflammatory amplification occurring within the atherosclerotic microenvironment [185].

While few direct causal studies have established a link between MD-induced alterations to the microbiome and decreased PAF activity in the circulation or vasculature, the combination of (i) improved gut barrier function and reduced endotoxin exposure; (ii) increased anti-inflammatory signals through SCFAs; and (iii) decreased prothrombotic microbial metabolite profiles provide a biologically plausible basis for an overall decrease in stimuli that promote PAF production and subsequent atherothrombotic events. Interestingly, the consumption of a yogurt fortified with OOPLE, containing PAF antagonists, led to a significant increase in the abundance of *Bifidobacterium* spp.—recognized as beneficial microorganisms—and a concomitant reduction in fecal caproic acid levels, a medium-chain fatty acid implicated in pro-inflammatory activity via the activation of the p38 MAPK signaling pathway in apparently healthy, predominantly overweight participants [205].

Furthermore, individual differences in response to the same dietary regimen are substantial, indicating that an individual’s baseline microbiota, medications (such as antibiotics, statins and metformin), and genetic background will all likely influence their response to the MD [206,207].

## 7. Lipid Composition vs. Phenolic Content: Reassessing the Protective Hierarchy of the MD

We propose that the athero-protective effects of the MD are primarily driven by its unique lipid composition rather than its phenolic content alone. This perspective is supported by the PREDIMED study, which demonstrated that MD patterns shift the systemic environment toward a more antioxidant profile compared to low-fat diets [86]. Furthermore, long-term adherence to the MD has been shown to halt the progression of atherosclerosis, evidenced by reduced carotid intima-media thickness (IMT-CC), smaller plaque height, and improved FMD compared to low-fat alternatives (CORDIOPREV Study) [208]. Crucially, animal models using hyperlipidemic rabbits revealed that the anti-atherogenic capacity of olive oil resides exclusively within the polar lipid fraction containing PAF inhibitors (glycerylether-glycolipids [5]). In contrast, the remaining fraction, despite containing triglycerides, phenols, and vitamins, failed to inhibit lesion formation in these models [209]. Nevertheless, the other minor constituents of olive oil have a synergistic role in its beneficial effects on atherogenesis and cardiovascular diseases [5].

Thus, the beneficial effects of the MD likely stem from a synergistic action of lipid-derived microconstituents. While high intake of MUFAs is linked to lower mortality, these benefits are predominantly associated with olive oil rather than animal-derived or other plant-derived MUFAs, as shown by a meta-analysis [210]. Mechanistic studies further indicate that the MD regulates inflammatory and postprandial coagulation responses, aided by synergy from nitrate-rich vegetables and moderate wine consumption [99,127]. This cumulative effect is underscored by findings that the MD’s association with reduced cardiovascular disease remains significant even after excluding individual metabolites like EPA or DHA [153].

Central to this lipid-driven protection is the modulation of the PAF pathway. Adoption of the MD significantly alters circulating glycerophospholipids, specifically phosphatidylcholines (PCs) and lysophosphatidylcholines (LysoPCs), which are direct precursors or metabolic relatives of PAF [154]. Although the extreme potency and low concentration of PAF have historically hindered its quantification in untargeted lipidomics, establishing affordable monitoring for PAF remains a priority for managing cardiovascular risk in other diseases, such as COVID-19 [211]. Evidence from rabbit models confirms that polar lipids (e.g., from fish) attenuate the progression of atherosclerosis specifically by down-regulating PAF biosynthesis and up-regulating its catabolism [212]. Ultimately, the pivotal role of PAF in thrombo-inflammatory processes—particularly in driving neutrophil activation and platelet interactions—elucidates the mechanism by which these specialized MD lipids provide superior vascular defense [213].

## 8. The Role of MD in Modulating PAF Synthesis and Activity: A Unifying Mechanistic Approach Against CVD

A plethora of research data suggests that the MD with EVOO as the main source of fat and adequate consumption of plant foods and fish leads to a reduction in the incidence of cardiovascular diseases and mortality [4,208]. Under this perspective, the question of whether there is a causal molecular factor to initiate atherosclerotic plaque formation has been the subject of many research groups during the last few decades. This protective effect is now thought to be due to the micro-constituents of the MD, including PAF inhibitors, highlighting a new scientific field of research. In this context, “the PAF-Implicated Atherosclerosis Theory” led to the rationale that natural compounds inhibiting PAF would have a protective effect on atherogenesis, cardiovascular diseases, and other diseases in which PAF is involved [61]. Indeed, it has been shown that the characteristic “healthy” MD foods, especially olive oil, wine, fish, onion, garlic, and vegetables, have PAF inhibitors that can favorably modify the pro-inflammatory actions of PAF and regulate its metabolism. This perspective has been evaluated in an extensive review exploring the correlation between MD and PAF [137].

### 8.1. PAF Inhibitors

PAF inhibitors with a glyceryl-ether glycolipid structure were initially identified in olive oil, where they were reported to exhibit inhibitory activity against PAF in vitro, acting as inhibitors or antagonists in washed rabbit platelets and in human platelet-rich plasma [214]. These compounds were subsequently detected in olive pomace [215]. Structurally related lipid species with reported anti-PAF activity were later identified in seed oils, although at considerably lower concentrations than those observed in olive oil; notably, these constituents appear to be largely removed during standard refining processes [214]. Subsequent investigations characterized the chemical structures of the principal bioactive compounds isolated from these sources [215].

In addition, PAF-inhibitory activity has been described in extracts derived from other foods characteristic of the MD, including fish polar lipid fractions, such as gangliosides [216,217] and diacyl- or alkyl-acyl- phosphatidyl choline and phoshatidyl ehtanolamine [218], honey [219], garlic [220], onion [221], egg yolk [222], and dairy [223]. Beyond lipid-derived molecules, micronutrients prevalent in the MD, particularly vitamins E and D, have also been associated with anti-PAF-related effects. Vitamin E has been reported to modulate platelet aggregation and to reduce in vitro plaque formation [224,225], while paricalcitol, a vitamin D analog, has been shown in hemodialysis patients to attenuate platelet aggregation, suppress PAF biosynthetic enzyme activity, and enhance the activity of the catabolic enzyme PAF-AH, thereby suggesting a potential mechanistic link between vitamin D signaling and PAF metabolism [226].

PAF inhibitors have been proposed as one potential biological explanation for the so-called “French Paradox,” a term introduced in 1992 to describe the epidemiological observation of relatively low coronary heart disease mortality in France despite a high intake of saturated fats [227,228]. The putative cardio-protective properties of wine have been largely attributed to its non-ethanolic microconstituents, prompting extensive efforts to isolate and characterize these bioactive compounds and to elucidate their underlying mechanisms of action. In vitro studies suggest that wine-derived microconstituents inhibit PAF-induced platelet aggregation [229,230] and modulate PAF biosynthesis in monocytes [231]. The chemical structures of the principal wine microconstituents exhibiting in vitro anti-PAF activity have been investigated and described, encompassing polar lipid fractions that also include polar phenolic compounds, such as resveratrol and tyrosol, which have been reported to exert PAF-inhibitory effects [232]. It should be emphasized, however, that contemporary public health guidance does not support alcohol consumption for cardio-protection. In this regard, the 2025 Scientific Statement from the American Heart Association underscores that alcohol intake at any level is associated with potential health risks and should not be recommended [233].

In addition, previous studies have reported the presence of PAF inhibitors in human blood and plasma, which represent the primary systemic compartment for circulating dietary nutrients and may play an important role in the pathophysiology of diseases such as coronary artery disease [234,235].

### 8.2. Constituents Related to Reduced PAF Levels

Antioxidants in MD foods partly prevent LDL oxidation and the formation of oxLDL containing PAF and oxidized lipids with PAF-like activity [62]. Findings from our group indicate that circulating PAF levels are inversely associated with both dietary antioxidant capacity and the consumption of antioxidant-dense foods, such as coffee and herbal infusions, even after adjusting for confounding variables [236]. Furthermore, a healthy dietary pattern characterized by high intakes of fruits, nuts, olive oil, and whole grains was linked to lower activity of Lyso-PAF-AT, the primary enzyme responsible for PAF biosynthesis [236].

Furthermore, resveratrol and tyrosol, two predominant phenolic constituents of wine and olive oil, have been reported to modulate PAF biosynthesis in monocytes. Specifically, both compounds have been shown to inhibit PAF production in interleukin-1β (IL-1β)-stimulated monocytes and to attenuate PAF biosynthetic activity in cell lysates. In unstimulated monocytes, resveratrol appears to exert a concentration-dependent, bidirectional effect on PAF biosynthesis: concentrations ≥50 μM were associated with a 20–43% reduction in the activity of PAF biosynthetic enzymes, whereas lower concentrations (10 μM) increased Ca^2+^-dependent lyso-PAF-AT (LysoPAF-ATC) activity by 28–45%, an effect mediated through activation of the p38 mitogen-activated protein kinase (p38 MAPK) pathway [237].

### 8.3. In Vivo Experiments with PAF Inhibitors

**Figure 3:**
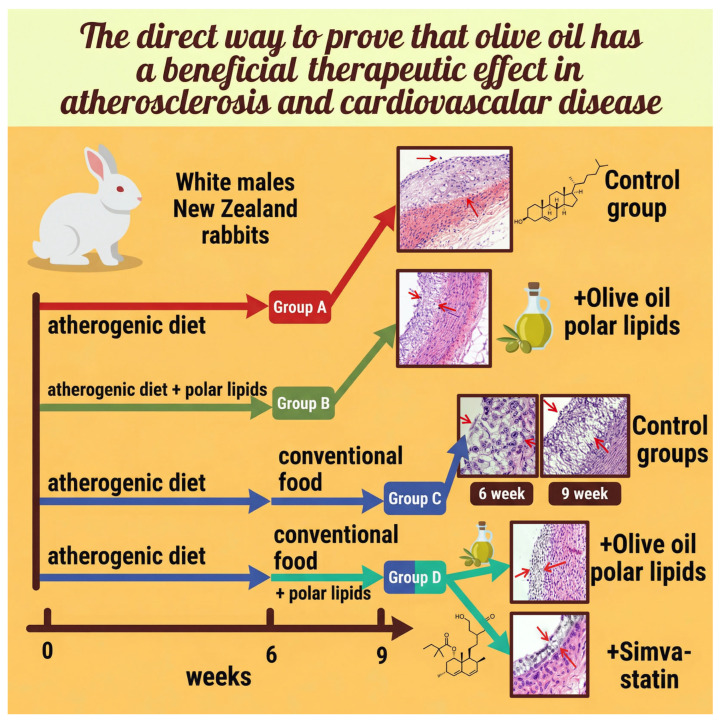
In vivo experiment showing the athero-protective and therapeutic effects of olive oil polar lipids, which act as PAF inhibitors. Figure Legend: Group A was fed a plain atherogenic diet (control group). Group B was fed an atherogenic diet enriched with olive oil polar lipids that contain PAF-antagonists and had reduced atherosclerosis lesions compared to Group A. Group C was fed an atherogenic diet and then conventional food (control group). Group D was fed an atherogenic diet and then conventional food + olive oil polar lipids or statin. Group D had a regression in the atherosclerotic plaque compared to Group C. Of note, the effects of olive oil polar lipids and statin were comparable. More details can be found in the relevant article of Tsantila et al. [239].

The anti-atherogenic effects of olive oil polar lipids have been demonstrated in rabbit models, in which either whole olive oil or its isolated polar lipid fraction was administered concomitantly with an atherogenic diet, resulting in marked inhibition of atherosclerotic plaque formation in both cases [238]. While animals fed a standard diet showed no plaque development, those receiving a high-cholesterol diet exhibited substantial atherogenesis; notably, co-administration of olive oil polar lipids effectively prevented plaque formation despite persistently elevated circulating cholesterol levels, suggesting a cholesterol-independent protective mechanism [238]. In regression experiments, the replacement of the atherogenic diet with standard feed, supplemented with either olive oil polar lipids or a statin, led to a measurable reduction in plaque thickness [239]. Plaque burden was closely associated with the presence of dietary PAF inhibitors in the circulation, as reflected by reduced PAF-induced platelet aggregability [239] (Figure 3).

These findings are consistent with accumulating evidence supporting a contributory role for PAF in atherogenesis that may be independent of circulating cholesterol levels [240]. Experimental support for this concept is further provided by studies demonstrating that administration of PAF inhibitors (including the PAF-specific ginkgolides derived from *Ginkgo biloba*) attenuates atheroma progression in hypercholesterolemic animal models [241,242].

Additional in vivo evidence is provided by studies in hypercholesterolemic rabbits fed an atherogenic diet enriched with a polar lipid extract from cultured gilthead sea bream (*Sparus aurata*), containing PAF antagonists [243]. Although lipid profiles (total cholesterol, LDL-C, and triacylglycerols) did not differ between control and enriched-diet groups, HDL-C levels were significantly higher, and atherogenesis was inhibited in animals receiving the polar-lipid-enriched diet. This protective effect was accompanied by modulation of PAF metabolism in leukocytes and platelets, including suppression of PAF biosynthetic enzyme activities and reduced circulating and cellular PAF levels, consistent with enhanced catabolic activity in plasma [212].

The protective effect of PAF inhibitors in atherogenesis is further supported by evidence demonstrating that ginkgolide B (BN 52021), a potent PAF antagonist, significantly reduces cholesterol accumulation in the atherosclerotic aorta of rabbits without altering plasma cholesterol levels. In addition, it ameliorates endothelial dysfunction and decreases inflammatory markers. Ginkgolide B has also been found to reduce markers of endothelial dysfunction (MCP-1, ICAM-1, and VCAM-1) in endothelial cells [244]. These findings suggest that ginkgolide B may represent a promising therapeutic agent for the prevention and management of atherosclerosis [240,244].

### 8.4. Human Intervention Studies

The first promising results from dietary intervention studies have shown that the administration of traditional [138] or fast-food MD meals [139] to either normal volunteers or type 2 diabetic patients (who have a predisposition to cardiovascular diseases) resulted in the characteristic lower PAF activity in blood (measured as PAF-induced platelet aggregability). In addition, intervention studies with functional foods, such as yogurt enriched with olive oil PAF inhibitors, or fish fed fish food containing olive oil PAF inhibitors (the PAF inhibitors were transferred to their flesh), on experimental animals and human volunteers confirm the beneficial effects of olive oil PAF inhibitors on atherosclerosis and cardiovascular disease [140].

In a randomized, double-blind crossover study, thirty healthy adults consumed both conventional fish and olive-pomace-enriched fish twice weekly over an eight-week period, separated by a six-week washout [245]. While the two interventions yielded largely similar results, the olive-pomace-enriched fish intake specifically led to increased PAF-CPT activity (PAF biosynthetic enzyme) and a reduction in arachidonic acid (AA) in red blood cell membranes [245].

In another study of our group, a functional yogurt, fortified with PAF inhibitors, was investigated in adults with overweight; in total, ninety-two adults were divided into three groups. Results showed that consuming the enriched yogurt significantly lowered interleukin-6 (IL-6) along with the activities of both PAF-CPT and Lp-PLA2, while the Lyso-PAF-AT isoforms remained unchanged. These findings suggest that a yogurt enriched with olive-derived inhibitors can beneficially modulate the biochemical pathways responsible for PAF synthesis and degradation [246].

In addition, a previous randomized postprandial clinical trial revealed that wine consumption along with a meal resulted in a reduction in platelet aggregation against PAF and decreased PAF biosynthetic enzyme activity compared to either ethanol or water alone, indicating that those effects are attributed to wine micro-constituents [125]. The above results support the notion that wine contains a mixture of micro-constituents in a proper quality and quantity that possess cardio-protective effects, partly through the modification of inflammation and thrombosis. Moreover, the light-to-moderate wine consumption for 8 weeks revealed an attenuation of the ethanol consumption effect on cytokine secretion at basal conditions from the patients’ peripheral blood mononuclear cells [247].

## 9. Conclusions

The complexity of CVDs requires a multifactorial approach to their prevention and management. In addition to genetics, which undoubtedly plays a role in CVD risk, lifestyle factors also contribute significantly to their occurrence and development, and of course, nutrition plays an important role. PAF inhibitors can inhibit early atherosclerosis development and explain in part the beneficial effects of MD. Whether PAF will eventually be formally categorized as an independent risk factor for atherosclerosis remains to be determined. However, the use of PAF inhibitors, including those found in MD, holds critical potential for the prevention and treatment of atherosclerosis and other inflammatory diseases.

## Figures and Tables

**Table 1:** Meta-analysis conducted in the last 15 years examining the effects of MD RCTs on inflammatory markers.

Studies per Marker	Total Included RCTs	Total Participants	Results
CRP			
[88]	11	1805	Mean Difference: −1.00 (−2.02, 0.01)
[89]	11	1035	Mean Difference: −0.83 (−1.25, −0.40)
[90]	16	3455	Standardized Mean Difference: −1.04 (−1.80, −0.27)
[91]	3	280	Standardized Mean Difference: −0.37 (−1.37, 0.64)
Interleukin-6			
[88]	8	524	Mean Difference −1.07 (−1.94, −0.20)
[90]	16	3455	Standardized Mean Difference: −0.38 (−0.55, −0.21)
Interleukin-1β			
[88]	3	178	Mean Difference: −0.49 (−0.66, −0.25)
Interleukin-8			
[88]	5	229	Mean Difference: −1.34 (−2.96, 0.14)
TNF-α			
[88]	4	335	Mean Difference: −1.69 (−3.40, 0.02)
[89]	3	234	Mean Difference: 0.17 (−0.38, 0.72)
[90]	16	3455	Standardized Mean Difference: −0.28 (−0.51, −0.05)

## Data Availability

No new data were created or analyzed in this study. Data sharing is not applicable to this article.

## Abbreviations

The following abbreviations are used in this manuscript:

CVD	cardiovascular disease
COX-2	Cyclooxygenase-2
IFN-γ	interferon-gamma
MMP-9	Matrix Metalloproteinase-9
LPS	Lipopolysaccharides
LysoPCs	lysophosphatidylcholines
MD	Mediterranean Diet
NADPH	Nicotinamide Adenine Dinucleotide Phosphate
NF-κB	nuclear factor κB
SCFAs	Short-chain fatty acids
TMAO	trimethylamine N-oxide
TMA	trimethylamine
PAF	Platelet-Activating Factor

## References

[1] Stark B.A., DeCleene N.K., Desai E.C., Hsu J.M., Johnson C.O., Lara-Castor L., LeGrand K.E., Bhoomadevi A., Aalipour M.A., Aalruz H. (2025). Global, Regional, and National Burden of Cardiovascular Diseases and Risk Factors in 204 Countries and Territories, 1990–2023. J. Am. Coll. Cardiol..

[2] Joseph P., Lanas F., Roth G., Lopez-Jaramillo P., Lonn E., Miller V., Mente A., Leong D., Schwalm J.-D., Yusuf S. (2025). Cardiovascular Disease in the Americas: The Epidemiology of Cardiovascular Disease and Its Risk Factors. Lancet Reg. Health Am..

[3] Visseren F.L.J., Mach F., Smulders Y.M., Carballo D., Koskinas K.C., Bäck M., Benetos A., Biffi A., Boavida J.-M., Capodanno D. (2021). 2021 ESC Guidelines on Cardiovascular Disease Prevention in Clinical Practice. Eur. Heart J..

[4] Menotti A., Puddu P.E. (2015). How the Seven Countries Study Contributed to the Definition and Development of the Mediterranean Diet Concept: A 50-Year Journey. Nutr. Metab. Cardiovasc. Dis..

[5] Antonopoulou S., Demopoulos C.A. (2023). Protective Effect of Olive Oil Microconstituents in Atherosclerosis: Emphasis on PAF Implicated Atherosclerosis Theory. Biomolecules.

[6] Davis C., Bryan J., Hodgson J., Murphy K. (2015). Definition of the Mediterranean Diet; A Literature Review. Nutrients.

[7] Detopoulou P., Aggeli M., Andrioti E., Detopoulou M. (2017). Macronutrient Content and Food Exchanges for 48 Greek Mediterranean Dishes. Nutr. Diet..

[8] Bozkurt O., Kocaadam Bozkurt B., Koçyiğit E. (2024). Evaluation of the Relationships Among Mindful Eating, Environmental Beliefs, Adherence to the Mediterranean Diet, and Obesity in Children. Turk. Arch. Pediatr..

[9] 8.COM Representative List of the Intangible Cultural Heritage of Humanity. https://Ich.Unesco.Org/En/RL/the-Mediterranean-Diet-00884.

[10] Sotos-Prieto M., Moreno-Franco B., Ordovás J.M., León M., Casasnovas J.A., Peñalvo J.L. (2015). Design and Development of an Instrument to Measure Overall Lifestyle Habits for Epidemiological Research: The Mediterranean Lifestyle (MEDLIFE) Index. Public. Health Nutr..

[11] Bôto J.M., Rocha A., Miguéis V., Meireles M., Neto B. (2022). Sustainability Dimensions of the Mediterranean Diet: A Systematic Review of the Indicators Used and Its Results. Adv. Nutr..

[12] World Health Organization (2019). Sustainable Healthy Diets.

[13] Sofi F., Martini D., Angelino D., Cairella G., Campanozzi A., Danesi F., Dinu M., Erba D., Iacoviello L., Pellegrini N. (2025). Mediterranean Diet: Why a New Pyramid? An Updated Representation of the Traditional Mediterranean Diet by the Italian Society of Human Nutrition (SINU). Nutr. Metab. Cardiovasc. Dis..

[14] Hutchins-Wiese H.L., Bales C.W., Porter Starr K.N. (2022). Mediterranean Diet Scoring Systems: Understanding the Evolution and Applications for Mediterranean and Non-Mediterranean Countries. Br. J. Nutr..

[15] Trichopoulou A., Costacou T., Bamia C., Trichopoulos D. (2003). Adherence to a Mediterranean Diet and Survival in a Greek Population. N. Engl. J. Med..

[16] Panagiotakos D.B., Pitsavos C., Stefanadis C. (2006). Dietary Patterns: A Mediterranean Diet Score and Its Relation to Clinical and Biological Markers of Cardiovascular Disease Risk. Nutr. Metab. Cardiovasc. Dis..

[17] Schröder H., Fitó M., Estruch R., Martínez-González M.A., Corella D., Salas-Salvadó J., Lamuela-Raventós R., Ros E., Salaverría I., Fiol M. (2011). A Short Screener Is Valid for Assessing Mediterranean Diet Adherence among Older Spanish Men and Women. J. Nutr..

[18] Detopoulou P., Dedes V., Syka D., Tzirogiannis K., Panoutsopoulos G.I. (2022). Mediterranean Diet, a Posteriori Dietary Patterns, Time-Related Meal Patterns and Adiposity: Results from a Cross-Sectional Study in University Students. Diseases.

[19] Veronese N., Gianfredi V., Volpe M., Zanetti M., Onder G., Silano M., Nucci D., Fontana L., Laviano A., Sieber C. (2026). 2025 National Guidelines on the Mediterranean Diet: Executive Summary of a Joint Report by Italian Scientific Societies and the National Institute of Health Task Force on Clinical Practice Guidelines. Nutr. Rev..

[20] Lichtenstein A.H., Appel L.J., Vadiveloo M., Hu F.B., Kris-Etherton P.M., Rebholz C.M., Sacks F.M., Thorndike A.N., Van Horn L., Wylie-Rosett J. (2021). 2021 Dietary Guidance to Improve Cardiovascular Health: A Scientific Statement From the American Heart Association. Circulation.

[21] McEvoy J.W., McCarthy C.P., Bruno R.M., Brouwers S., Canavan M.D., Ceconi C., Christodorescu R.M., Daskalopoulou S.S., Ferro C.J., Gerdts E. (2024). 2024 ESC Guidelines for the Management of Elevated Blood Pressure and Hypertension. Eur. Heart J..

[22] Bajaj M., McCoy R.G., Balapattabi K., Bannuru R.R., Bellini N.J., Bennett A.K., Beverly E.A., Briggs Early K., ChallaSivaKanaka S., American Diabetes Association Professional Practice Committee for Diabetes (2026). 1. Improving Care and Promoting Health in Populations: Standards of Care in Diabetes—2026. Diabetes Care.

[23] Aas A.-M., Axelsen M., Churuangsuk C., Hermansen K., Kendall C.W.C., Kahleova H., Khan T., Lean M.E.J., Mann J.I., The Diabetes and Nutrition Study Group (DNSG) of the European Association for the Study of Diabetes (EASD) (2023). Evidence-Based European Recommendations for the Dietary Management of Diabetes. Diabetologia.

[24] Verschuren W.M., Jacobs D.R., Bloemberg B.P., Kromhout D., Menotti A., Aravanis C., Blackburn H., Buzina R., Dontas A.S., Fidanza F. (1995). Serum Total Cholesterol and Long-Term Coronary Heart Disease Mortality in Different Cultures. Twenty-Five-Year Follow-up of the Seven Countries Study. JAMA.

[25] Simini B. (2000). Serge Renaud: From French Paradox to Cretan Miracle. Lancet.

[26] Keys A., Keys M. (1959). Eat Well and Stay Well.

[27] Kris-Etherton P., Eckel R.H., Howard B.V., St. Jeor S., Bazzarre T.L. (2001). Lyon Diet Heart Study: Benefits of a Mediterranean-Style, National Cholesterol Education Program/American Heart Association Step I Dietary Pattern on Cardiovascular Disease. Circulation.

[28] Estruch R., Ros E., Salas-Salvadó J., Covas M.-I., Corella D., Arós F., Gómez-Gracia E., Ruiz-Gutiérrez V., Fiol M., Lapetra J. (2018). Primary Prevention of Cardiovascular Disease with a Mediterranean Diet Supplemented with Extra-Virgin Olive Oil or Nuts. N. Engl. J. Med..

[29] Delgado-Lista J., Alcala-Diaz J.F., Torres-Peña J.D., Quintana-Navarro G.M., Fuentes F., Garcia-Rios A., Ortiz-Morales A.M., Gonzalez-Requero A.I., Perez-Caballero A.I., Yubero-Serrano E.M. (2022). Long-Term Secondary Prevention of Cardiovascular Disease with a Mediterranean Diet and a Low-Fat Diet (CORDIOPREV): A Randomised Controlled Trial. Lancet.

[30] Pant A., Gribbin S., McIntyre D., Trivedi R., Marschner S., Laranjo L., Mamas M.A., Flood V., Chow C.K., Zaman S. (2023). Primary Prevention of Cardiovascular Disease in Women with a Mediterranean Diet: Systematic Review and Meta-Analysis. Heart.

[31] Volpe R., Ciccone M.M., Pala B., Barbarano F., Camastra S., Caprio M., Casirati A., Ferrera A., Galletti F., Greatti A. (2026). Efficacy of Mediterranean Diet for the Prevention in Patients Affected by Cardiovascular Diseases: A Systematic Review and Meta-Analysis Featured in the Italian National Guidelines “La Dieta Mediterranea”. Nutrition.

[32] Sebastian S.A., Padda I., Johal G. (2024). Long-Term Impact of Mediterranean Diet on Cardiovascular Disease Prevention: A Systematic Review and Meta-Analysis of Randomized Controlled Trials. Curr. Probl. Cardiol..

[33] Sofi F., Macchi C., Abbate R., Gensini G.F., Casini A. (2014). Mediterranean Diet and Health Status: An Updated Meta-Analysis and a Proposal for a Literature-Based Adherence Score. Public Health Nutr..

[34] Doundoulakis I., Farmakis I.T., Theodoridis X., Konstantelos A., Christoglou M., Kotzakioulafi E., Chrysoula L., Siargkas A., Karligkiotis A., Kyprianou G. (2024). Effects of Dietary Interventions on Cardiovascular Outcomes: A Network Meta-Analysis. Nutr. Rev..

[35] Reyneke G.L., Lambert K., Beck E.J. (2025). Dietary Patterns Associated With Anti-Inflammatory Effects: An Umbrella Review of Systematic Reviews and Meta-Analyses. Nutr. Rev..

[36] Dinu M., Pagliai G., Casini A., Sofi F. (2018). Mediterranean Diet and Multiple Health Outcomes: An Umbrella Review of Meta-Analyses of Observational Studies and Randomised Trials. Eur. J. Clin. Nutr..

[37] Grosso G., Marventano S., Yang J., Micek A., Pajak A., Scalfi L., Galvano F., Kales S.N. (2017). A Comprehensive Meta-Analysis on Evidence of Mediterranean Diet and Cardiovascular Disease: Are Individual Components Equal?. Crit. Rev. Food Sci. Nutr..

[38] Martínez-González M.A., Sayón-Orea C., Bullón-Vela V., Bes-Rastrollo M., Rodríguez-Artalejo F., Yusta-Boyo M.J., García-Solano M. (2022). Effect of Olive Oil Consumption on Cardiovascular Disease, Cancer, Type 2 Diabetes, and All-Cause Mortality: A Systematic Review and Meta-Analysis. Clin. Nutr..

[39] Lordan R., Tsoupras A., Zabetakis I., Demopoulos C.A. (2019). Forty Years Since the Structural Elucidation of Platelet-Activating Factor (PAF): Historical, Current, and Future Research Perspectives. Molecules.

[40] Demopoulos C.A., Pinckard R.N., Hanahan D.J. (1979). Platelet-Activating Factor. Evidence for 1-O-Alkyl-2-Acetyl-Sn-Glyceryl-3-Phosphorylcholine as the Active Component (a New Class of Lipid Chemical Mediators). J. Biol. Chem..

[41] Demopoulos C.A. (2025). The Story Behind the Discovery of the Structure of Platelet-Activating Factor: A Potent Lipid Inflammatory Mediator. J. Atheroscler. Prev. Treat..

[42] Prescott S.M., Zimmerman G.A., Stafforini D.M., McIntyre T.M. (2000). Platelet-Activating Factor and Related Lipid Mediators. Annu. Rev. Biochem..

[43] Pike I., Ammit A., O’Neill C. (1992). Actions of Platelet Activating Factor (PAF) on Gametes and Embryos: Clinical Aspects. Reprod. Fertil. Dev..

[44] Pantazi D., Tellis C., Tselepis A.D. (2022). Oxidized Phospholipids and Lipoprotein-Associated Phospholipase A2 (Lp-PLA2) in Atherosclerotic Cardiovascular Disease: An Update. Biofactors.

[45] Pinckard R.N., Farr R.S., Hanahan D.J. (1979). Physicochemical and Functional Identity of Rabbit Platelet-Activating Factor (PAF) Released in Vivo during IgE Anaphylaxis with PAF Released in Vitro from IgE Sensitized Basophils. J. Immunol..

[46] Kelesidis T., Papakonstantinou V., Detopoulou P., Fragopoulou E., Chini M., Lazanas M.C., Antonopoulou S. (2015). The Role of Platelet-Activating Factor in Chronic Inflammation, Immune Activation, and Comorbidities Associated with HIV Infection. AIDS Rev..

[47] Detopoulou P., Nomikos T., Fragopoulou E., Chrysohoou C., Antonopoulou S. (2013). Platelet Activating Factor in Heart Failure: Potential Role in Disease Progression and Novel Target for Therapy. Curr. Heart Fail. Rep..

[48] Detopoulou P., Demopoulos C.A., Antonopoulou S. (2021). Micronutrients, Phytochemicals and Mediterranean Diet: A Potential Protective Role against COVID-19 through Modulation of PAF Actions and Metabolism. Nutrients.

[49] Tsoupras A., Adamantidi T., Finos M.A., Philippopoulos A., Detopoulou P., Tsopoki I., Kynatidou M., Demopoulos C.A. (2024). Re-Assessing the Role of Platelet Activating Factor and Its Inflammatory Signaling and Inhibitors in Cancer and Anti-Cancer Strategies. Front. Biosci..

[50] Tsoupras A., Iatrou C., Frangia C., Demopoulos C. (2009). The Implication of Platelet Activating Factor in Cancer Growth and Metastasis: Potent Beneficial Role of PAF-Inhibitors and Antioxidants. Infect. Disord. Drug Targets.

[51] Negro Alvarez J.M., Miralles López J.C., Ortiz Martínez J.L., Abellán Alemán A., Rubio del Barrio R. (1997). Platelet-Activating Factor Antagonists. Allergol. Immunopathol..

[52] Merlos M., Giral M., Balsa D., Ferrando R., Queralt M., Puigdemont A., García-Rafanell J., Forn J. (1997). Rupatadine, a New Potent, Orally Active Dual Antagonist of Histamine and Platelet-Activating Factor (PAF). J. Pharmacol. Exp. Ther..

[53] Tsantila N., Tsoupras A.B., Fragopoulou E., Antonopoulou S., Iatrou C., Demopoulos C.A. (2011). In Vitro and In Vivo Effects of Statins on Platelet-Activating Factor and Its Metabolism. Angiology.

[54] Tsoupras A.B., Chini M., Tsogas N., Fragopoulou E., Nomikos T., Lioni A., Mangafas N., Demopoulos C.A., Antonopoulou S., Lazanas M.C. (2008). Anti-Platelet-Activating Factor Effects of Highly Active Antiretroviral Therapy (HAART): A New Insight in the Drug Therapy of HIV Infection?. AIDS Res. Hum. Retroviruses.

[55] Moschos M.M., Moustafa G.A., Papakonstantinou V.D., Tsatsos M., Laios K., Antonopoulou S. (2017). Anti-Platelet Effects of Anti-Glaucomatous Eye Drops: An in Vitro Study on Human Platelets. Drug Des. Devel. Ther..

[56] Ross R., Glomset J., Harker L. (1977). Response to Injury and Atherogenesis. Am. J. Pathol..

[57] Brown M.S., Goldstein J.L. (1983). Lipoprotein metabolism in the macrophage: Implications for Cholesterol Deposition in Atherosclerosis. Annu. Rev. Biochem..

[58] Bolanle I.O., de Liedekerke Beaufort G.C., Weinberg P.D. (2025). Transcytosis of LDL Across Arterial Endothelium: Mechanisms and Therapeutic Targets. Arter. Thromb. Vasc. Biol..

[59] Poznyak A.V., Nikiforov N.G., Markin A.M., Kashirskikh D.A., Myasoedova V.A., Gerasimova E.V., Orekhov A.N. (2021). Overview of OxLDL and Its Impact on Cardiovascular Health: Focus on Atherosclerosis. Front. Pharmacol..

[60] Heery J.M., Kozak M., Stafforini D.M., Jones D.A., Zimmerman G.A., McIntyre T.M., Prescott S.M. (1995). Oxidatively Modified LDL Contains Phospholipids with Platelet-Activating Factor-like Activity and Stimulates the Growth of Smooth Muscle Cells. J. Clin. Invest..

[61] Demopoulos C.A., Karantonis H.C., Antonopoulou S. (2003). Platelet Activating Factor—A Molecular Link between Atherosclerosis Theories. Eur. J. Lipid Sci. Technol..

[62] Liapikos T.A., Antonopoulou S., Karabina S.-A.P., Tsoukatos D.C., Demopoulos C.A., Tselepis A.D. (1994). Platelet-Activating Factor Formation during Oxidative Modification of Low-Density Lipoprotein When PAF-Acetylhydrolase Has Been Inactivated. Biochim. Et. Biophys. Acta (BBA) Lipids Lipid Metab..

[63] Keizer H.G. (2012). The “Mevalonate Hypothesis”: A Cholesterol-Independent Alternative for the Etiology of Atherosclerosis. Lipids Health Dis..

[64] Jebari-Benslaiman S., Galicia-García U., Larrea-Sebal A., Olaetxea J.R., Alloza I., Vandenbroeck K., Benito-Vicente A., Martín C. (2022). Pathophysiology of Atherosclerosis. Int. J. Mol. Sci..

[65] Ed Rainger G., Chimen M., Harrison M.J., Yates C.M., Harrison P., Watson S.P., Lordkipanidzé M., Nash G.B. (2015). The Role of Platelets in the Recruitment of Leukocytes during Vascular Disease. Platelets.

[66] Palur Ramakrishnan A.V.K., Varghese T.P., Vanapalli S., Nair N.K., Mingate M.D. (2017). Platelet Activating Factor: A Potential Biomarker in Acute Coronary Syndrome?. Cardiovasc. Ther..

[67] Ninio E. (2005). Phospholipid Mediators in the Vessel Wall: Involvement in Atherosclerosis. Curr. Opin. Clin. Nutr. Metab. Care.

[68] Zimmerman G.A., Elstad M.R., Lorant D.E., Mclntyre T.M., Prescott S.M., Topham M.K., Weyrich A.S., Whatley R.E. (1996). Platelet-Activating Factor (PAF): Signalling and Adhesion in Cell-Cell Interactions. Platelet-Activating Factor and Related Lipid Mediators 2.

[69] Yang Y., Yin J., Baumgartner W., Samapati R., Solymosi E.A., Reppien E., Kuebler W.M., Uhlig S. (2010). Platelet-Activating Factor Reduces Endothelial Nitric Oxide Production: Role of Acid Sphingomyelinase. Eur. Respir. J..

[70] Bussolino F., Camussi G. (1995). Platelet-Activating Factor Produced by Endothelial Cells. A Molecule with Autocrine and Paracrine Properties. Eur. J. Biochem..

[71] Verouti S.N., Fragopoulou E., Karantonis H.C., Dimitriou A.A., Tselepis A.D., Antonopoulou S., Nomikos T., Demopoulos C.A. (2011). PAF Effects on MCP-1 and IL-6 Secretion in U-937 Monocytes in Comparison with oxLDL and IL-1β Effects. Atherosclerosis.

[72] Huang Y.H., Schäfer-Elinder L., Owman H., Lorentzen J.C., Rönnelid J., Frostegård J. (1996). Induction of IL-4 by Platelet-Activating Factor. Clin. Exp. Immunol..

[73] Marathe G.K., Harrison K.A., Murphy R.C., Prescott S.M., Zimmerman G.A., McIntyre T.M. (2000). Bioactive Phospholipid Oxidation Products. Free Radic. Biol. Med..

[74] Chen K., Sun W., Jiang Y., Chen B., Zhao Y., Sun J., Gong H., Qi R. (2017). Ginkgolide B Suppresses TLR4-Mediated Inflammatory Response by Inhibiting the Phosphorylation of JAK2/STAT3 and P38 MAPK in High Glucose-Treated HUVECs. Oxid. Med. Cell Longev..

[75] Kong P., Cui Z.-Y., Huang X.-F., Zhang D.-D., Guo R.-J., Han M. (2022). Inflammation and Atherosclerosis: Signaling Pathways and Therapeutic Intervention. Signal Transduct. Target. Ther..

[76] Tsimikas S., Witztum J.L. (2024). Oxidized Phospholipids in Cardiovascular Disease. Nat. Rev. Cardiol..

[77] Tsironis L.D., Katsouras C.S., Lourida E.S., Mitsios J.V., Goudevenos J., Elisaf M., Tselepis A.D. (2004). Reduced PAF-Acetylhydrolase Activity Associated with Lp(a) in Patients with Coronary Artery Disease. Atherosclerosis.

[78] Aviram M., Fuhrman B. (1998). Polyphenolic Flavonoids Inhibit Macrophage-Mediated Oxidation of LDL and Attenuate Atherogenesis. Atherosclerosis.

[79] EFSA Panel on Dietetic Products, Nutrition and Allergies (NDA) (2011). Scientific Opinion on the Substantiation of Health Claims Related to Polyphenols in Olive and Protection of LDL Particles from Oxidative Damage (ID 1333, 1638, 1639, 1696, 2865), Maintenance of Normal Blood HDL-Cholesterol Concentrations (ID 1639), Maintenance of Normal Blood Pressure (ID 3781), “Anti-Inflammatory Properties” (ID 1882), “Contributes to the Upper Respiratory Tract Health” (ID 3468), “Can Help to Maintain a Normal Function of Gastrointestinal Tract” (3779), and “Contributes to Body Defences against External Agents” (ID 3467) Pursuant to Article 13(1) of Regulation (EC) No 1924/2006. EFSA J..

[80] Martínez-Huélamo M., Rodríguez-Morató J., Boronat A., De La Torre R. (2017). Modulation of Nrf2 by Olive Oil and Wine Polyphenols and Neuroprotection. Antioxidants.

[81] Boocock D.J., Faust G.E.S., Patel K.R., Schinas A.M., Brown V.A., Ducharme M.P., Booth T.D., Crowell J.A., Perloff M., Gescher A.J. (2007). Phase I Dose Escalation Pharmacokinetic Study in Healthy Volunteers of Resveratrol, a Potential Cancer Chemopreventive Agent. Cancer Epidemiol. Biomark. Prev..

[82] Ungvari Z., Bagi Z., Feher A., Recchia F.A., Sonntag W.E., Pearson K., De Cabo R., Csiszar A. (2010). Resveratrol Confers Endothelial Protection via Activation of the Antioxidant Transcription Factor Nrf2. Am. J. Physiol. Heart Circ. Physiol..

[83] Ghiselli A., D’Amicis A., Giacosa A. (1997). The Antioxidant Potential of the Mediterranean Diet. Eur. J. Cancer Prev..

[84] Ninfali P., Mea G., Giorgini S., Rocchi M., Bacchiocca M. (2005). Antioxidant Capacity of Vegetables, Spices and Dressings Relevant to Nutrition. Br. J. Nutr..

[85] Lozano-Castellón J., Rinaldi De Alvarenga J.F., Vallverdú-Queralt A., Lamuela-Raventós R.M. (2022). Cooking with Extra-Virgin Olive Oil: A Mixture of Food Components to Prevent Oxidation and Degradation. Trends Food Sci. Technol..

[86] Razquin C., Martinez J.A., Martinez-Gonzalez M.A., Mitjavila M.T., Estruch R., Marti A. (2009). A 3 Years Follow-up of a Mediterranean Diet Rich in Virgin Olive Oil Is Associated with High Plasma Antioxidant Capacity and Reduced Body Weight Gain. Eur. J. Clin. Nutr..

[87] Fitó M., Guxens M., Corella D., Sáez G., Estruch R., De La Torre R., Francés F., Cabezas C., López-Sabater M.D.C., Marrugat J. (2007). Effect of a Traditional Mediterranean Diet on Lipoprotein Oxidation: A Randomized Controlled Trial. Arch. Intern. Med..

[88] Koelman L., Egea Rodrigues C., Aleksandrova K. (2022). Effects of Dietary Patterns on Biomarkers of Inflammation and Immune Responses: A Systematic Review and Meta-Analysis of Randomized Controlled Trials. Adv. Nutr..

[89] Neale E.P., Batterham M.J., Tapsell L.C. (2016). Consumption of a Healthy Dietary Pattern Results in Significant Reductions in C-Reactive Protein Levels in Adults: A Meta-Analysis. Nutr. Res..

[90] Pourrajab B., Fotros D., Asghari P., Shidfar F. (2025). Effect of the Mediterranean Diet Supplemented With Olive Oil Versus the Low-Fat Diet on Serum Inflammatory and Endothelial Indexes Among Adults: A Systematic Review and Meta-Analysis of Clinical Controlled Trials. Nutr. Rev..

[91] Sánchez-Rosales A.I., Guadarrama-López A.L., Gaona-Valle L.S., Martínez-Carrillo B.E., Valdés-Ramos R. (2022). The Effect of Dietary Patterns on Inflammatory Biomarkers in Adults with Type 2 Diabetes Mellitus: A Systematic Review and Meta-Analysis of Randomized Controlled Trials. Nutrients.

[92] Nani A., Murtaza B., Sayed Khan A., Khan N.A., Hichami A. (2021). Antioxidant and Anti-Inflammatory Potential of Polyphenols Contained in Mediterranean Diet in Obesity: Molecular Mechanisms. Molecules.

[93] Scoditti E., Calabriso N., Massaro M., Pellegrino M., Storelli C., Martines G., De Caterina R., Carluccio M.A. (2012). Mediterranean Diet Polyphenols Reduce Inflammatory Angiogenesis through MMP-9 and COX-2 Inhibition in Human Vascular Endothelial Cells: A Potentially Protective Mechanism in Atherosclerotic Vascular Disease and Cancer. Arch. Biochem. Biophys..

[94] Moreno-Luna R., Muñoz-Hernandez R., Miranda M.L., Costa A.F., Jimenez-Jimenez L., Vallejo-Vaz A.J., Muriana F.J.G., Villar J., Stiefel P. (2012). Olive Oil Polyphenols Decrease Blood Pressure and Improve Endothelial Function in Young Women with Mild Hypertension. Am. J. Hypertens..

[95] Lordan R., Redfern S., Tsoupras A., Zabetakis I. (2020). Inflammation and Cardiovascular Disease: Are Marine Phospholipids the Answer?. Food Funct..

[96] Calder P.C. (2017). Omega-3 Fatty Acids and Inflammatory Processes: From Molecules to Man. Biochem. Soc. Trans..

[97] Sperling R.I., Simopoulos A.P., Kifer R.R., Martin R.E., Barlow S. (1991). Effects of Dietary Fish Oil on Leukocyte Leukotriene and PAF Generation and on Neutrophil Chemotaxis1. World Review of Nutrition and Dietetics.

[98] Kimble R., Gouinguenet P., Ashor A., Stewart C., Deighton K., Matu J., Griffiths A., Malcomson F.C., Joel A., Houghton D. (2023). Effects of a Mediterranean Diet on the Gut Microbiota and Microbial Metabolites: A Systematic Review of Randomized Controlled Trials and Observational Studies. Crit. Rev. Food Sci. Nutr..

[99] Capurso C., Massaro M., Scoditti E., Vendemiale G., Capurso A. (2014). Vascular Effects of the Mediterranean Diet Part I: Anti-Hypertensive and Anti-Thrombotic Effects. Vasc. Pharmacol..

[100] Castañer O., Covas M.-I., Khymenets O., Nyyssonen K., Konstantinidou V., Zunft H.-F., De La Torre R., Muñoz-Aguayo D., Vila J., Fitó M. (2012). Protection of LDL from Oxidation by Olive Oil Polyphenols Is Associated with a Downregulation of CD40-Ligand Expression and Its Downstream Products in Vivo in Humans. Am. J. Clin. Nutr..

[101] Rallidis L.S., Lekakis J., Kolomvotsou A., Zampelas A., Vamvakou G., Efstathiou S., Dimitriadis G., Raptis S.A., Kremastinos D.T. (2009). Close Adherence to a Mediterranean Diet Improves Endothelial Function in Subjects with Abdominal Obesity. Am. J. Clin. Nutr..

[102] Fuentes F., López-Miranda J., Sánchez E., Sánchez F., Paez J., Paz-Rojas E., Marín C., Gómez P., Jimenez-Perepérez J., Ordovás J.M. (2001). Mediterranean and Low-Fat Diets Improve Endothelial Function in Hypercholesterolemic Men. Ann. Intern. Med..

[103] Yubero-Serrano E.M., Fernandez-Gandara C., Garcia-Rios A., Rangel-Zuñiga O.A., Gutierrez-Mariscal F.M., Torres-Peña J.D., Marin C., Lopez-Moreno J., Castaño J.P., Delgado-Lista J. (2020). Mediterranean Diet and Endothelial Function in Patients with Coronary Heart Disease: An Analysis of the CORDIOPREV Randomized Controlled Trial. PLoS Med..

[104] Ambring A., Friberg P., Axelsen M., Laffrenzen M., Taskinen M.-R., Basu S., Johansson M. (2004). Effects of a Mediterranean-Inspired Diet on Blood Lipids, Vascular Function and Oxidative Stress in Healthy Subjects. Clin. Sci..

[105] Marin C., Ramirez R., Delgado-Lista J., Yubero-Serrano E.M., Perez-Martinez P., Carracedo J., Garcia-Rios A., Rodriguez F., Gutierrez-Mariscal F.M., Gomez P. (2011). Mediterranean Diet Reduces Endothelial Damage and Improves the Regenerative Capacity of Endothelium. Am. J. Clin. Nutr..

[106] Shannon O.M., Mendes I., KÖchl C., Mazidi M., Ashor A.W., Rubele S., Minihane A.-M., Mathers J.C., Siervo M. (2020). Mediterranean Diet Increases Endothelial Function in Adults: A Systematic Review and Meta-Analysis of Randomized Controlled Trials. J. Nutr..

[107] Karatzi K., Papamichael C., Karatzis E., Papaioannou T.G., Voidonikola P.T., Vamvakou G.D., Lekakis J., Zampelas A. (2008). Postprandial Improvement of Endothelial Function by Red Wine and Olive Oil Antioxidants: A Synergistic Effect of Components of the Mediterranean Diet. J. Am. Coll. Nutr..

[108] Schwingshackl L., Hoffmann G. (2014). Mediterranean Dietary Pattern, Inflammation and Endothelial Function: A Systematic Review and Meta-Analysis of Intervention Trials. Nutr. Metab. Cardiovasc. Dis..

[109] Meessen E.C.E., Warmbrunn M.V., Nieuwdorp M., Soeters M.R. (2019). Human Postprandial Nutrient Metabolism and Low-Grade Inflammation: A Narrative Review. Nutrients.

[110] Jacome-Sosa M., Parks E.J., Bruno R.S., Tasali E., Lewis G.F., Schneeman B.O., Rains T.M. (2016). Postprandial Metabolism of Macronutrients and Cardiometabolic Risk: Recent Developments, Emerging Concepts, and Future Directions. Adv. Nutr..

[111] Sloth B., Due A., Larsen T.M., Holst J.J., Heding A., Astrup A. (2008). The Effect of a High-MUFA, Low-Glycaemic Index Diet and a Low-Fat Diet on Appetite and Glucose Metabolism during a 6-Month Weight Maintenance Period. Br. J. Nutr..

[112] Paniagua J.A., De La Sacristana A.G., Sánchez E., Romero I., Vidal-Puig A., Berral F.J., Escribano A., Moyano M.J., Peréz-Martinez P., López-Miranda J. (2007). A MUFA-Rich Diet Improves Posprandial Glucose, Lipid and GLP-1 Responses in Insulin-Resistant Subjects. J. Am. Coll. Nutr..

[113] Defoort C., Vincent-Baudry S., Lairon D. (2011). Effects of 3-Month Mediterranean-Type Diet on Postprandial TAG and Apolipoprotein B48 in the Medi-RIVAGE Cohort. Public. Health Nutr..

[114] Perez-Martinez P., Ordovas J.M., Garcia-Rios A., Delgado-Lista J., Delgado-Casado N., Cruz-Teno C., Camargo A., Yubero-Serrano E.M., Rodriguez F., Perez-Jimenez F. (2011). Consumption of Diets with Different Type of Fat Influences Triacylglycerols-Rich Lipoproteins Particle Number and Size during the Postprandial State. Nutr. Metab. Cardiovasc. Dis..

[115] Leighton F., Urquiaga L., Smith J., Charter E. (2010). The Mediterranean Diets: Nutrition and Gastronomy. Functional Food Product Development.

[116] O’Keefe J.H., Gheewala N.M., O’Keefe J.O. (2008). Dietary Strategies for Improving Post-Prandial Glucose, Lipids, Inflammation, and Cardiovascular Health. J. Am. Coll. Cardiol..

[117] Riccardi G., Clemente G., Giacco R. (2003). Glycemic Index of Local Foods and Diets: The Mediterranean Experience. Nutr. Rev..

[118] Detopoulou P., Voulgaridou G., Seva V., Kounetakis O., Desli I.-I., Tsoumana D., Dedes V., Papachristou E., Papadopoulou S., Panoutsopoulos G. (2024). Dietary Restriction of Advanced Glycation End-Products (AGEs) in Patients with Diabetes: A Systematic Review of Randomized Controlled Trials. Int. J. Mol. Sci..

[119] Cruz N., Flores M., Urquiaga I., Ávila F. (2022). Modulation of 1,2-Dicarbonyl Compounds in Postprandial Responses Mediated by Food Bioactive Components and Mediterranean Diet. Antioxidants.

[120] Tsoupras A., Brummell C., Kealy C., Vitkaitis K., Redfern S., Zabetakis I. (2022). Cardio-Protective Properties and Health Benefits of Fish Lipid Bioactives; The Effects of Thermal Processing. Mar. Drugs.

[121] Pacheco Y.M., Bermúdez B., López S., Abia R., Villar J., Muriana F.J.G. (2007). Minor Compounds of Olive Oil Have Postprandial Anti-Inflammatory Effects. Br. J. Nutr..

[122] Lopez S., Bermudez B., Ortega A., Varela L.M., Pacheco Y.M., Villar J., Abia R., Muriana F.J. (2011). Effects of Meals Rich in Either Monounsaturated or Saturated Fat on Lipid Concentrations and on Insulin Secretion and Action in Subjects with High Fasting Triglyceride Concentrations. Am. J. Clin. Nutr..

[123] Park Y., Harris W.S. (2003). Omega-3 Fatty Acid Supplementation Accelerates Chylomicron Triglyceride Clearance. J. Lipid Res..

[124] Nomikos T., Detopoulou P., Fragopoulou E., Pliakis E., Antonopoulou S. (2007). Boiled Wild Artichoke Reduces Postprandial Glycemic and Insulinemic Responses in Normal Subjects but Has No Effect on Metabolic Syndrome Patients. Nutr. Res..

[125] Xanthopoulou M.N., Kalathara K., Melachroinou S., Arampatzi-Menenakou K., Antonopoulou S., Yannakoulia M., Fragopoulou E. (2017). Wine Consumption Reduced Postprandial Platelet Sensitivity against Platelet Activating Factor in Healthy Men. Eur. J. Nutr..

[126] Hendriks H.F.J., Veenstra J., Van Tol A., Groener J.E.M., Schaafsma G. (1998). Moderate Doses of Alcoholic Beverages with Dinner and Postprandial High Density Lipoprotein Composition. Alcohol. Alcohol..

[127] Mezzano D., Leighton F., Martínez C., Marshall G., Cuevas A., Castillo O., Panes O., Muñoz B., Pérez D., Mizón C. (2001). Complementary Effects of Mediterranean Diet and Moderate Red Wine Intake on Haemostatic Cardiovascular Risk Factors. Eur. J. Clin. Nutr..

[128] Fragopoulou E., Detopoulou P., Nomikos T., Pliakis E., Panagiotakos D.B., Antonopoulou S. (2012). Mediterranean Wild Plants Reduce Postprandial Platelet Aggregation in Patients with Metabolic Syndrome. Metabolism.

[129] Carter S.J., Roberts M.B., Salter J., Eaton C.B. (2010). Relationship between Mediterranean Diet Score and Atherothrombotic Risk: Findings from the Third National Health and Nutrition Examination Survey (NHANES III), 1988–1994. Atherosclerosis.

[130] Chrysohoou C., Panagiotakos D.B., Pitsavos C., Das U.N., Stefanadis C. (2004). Adherence to the Mediterranean Diet Attenuates Inflammation and Coagulation Process in Healthy Adults. J. Am. Coll. Cardiol..

[131] Di Castelnuovo A., Bonaccio M., De Curtis A., Costanzo S., Persichillo M., De Gaetano G., Donati M.B., Iacoviello L. (2017). Higher Adherence to the Mediterranean Diet Is Associated with Lower Levels of D-Dimer: Findings from the MOLI-SANI Study. Haematologica.

[132] Passaro A., Calzavarini S., Volpato S., Caruso P., Poli A., Fellin R., Bernardi F. (2008). Reduced Factor VII and Factor VIII Levels and Prolonged Thrombin-generation Times during a Healthy Diet in Middle-aged Women with Mild to Moderate Cardiovascular Disease Risk. J. Thromb. Haemost..

[133] De La Cruz J.P., Villalobos M.A., Carmona J.A., Martín-Romero M., Smith-Agreda J.M., De La Cuesta F.S. (2000). Antithrombotic Potential of Olive Oil Administration in Rabbits with Elevated Cholesterol. Thromb. Res..

[134] Renaud S., Morazain R., Godsey F., Dumont E., Thevenon C., Martin J.L., Mendy F. (1986). Nutrients, Platelet Function and Composition in Nine Groups of French and British Farmers. Atherosclerosis.

[135] Hernáez Á., Castañer O., Tresserra-Rimbau A., Pintó X., Fitó M., Casas R., Martínez-González M.Á., Corella D., Salas-Salvadó J., Lapetra J. (2020). Mediterranean Diet and Atherothrombosis Biomarkers: A Randomized Controlled Trial. Mol. Nutr. Food Res..

[136] Tohti I., Tursun M., Umar A., Turdi S., Imin H., Moore N. (2006). Aqueous Extracts of *Ocimum basilicum* L. (Sweet Basil) Decrease Platelet Aggregation Induced by ADP and Thrombin in Vitro and Rats Arterio–Venous Shunt Thrombosis in Vivo. Thromb. Res..

[137] Nomikos T., Fragopoulou E., Antonopoulou S., Panagiotakos D.B. (2018). Mediterranean Diet and Platelet-Activating Factor; a Systematic Review. Clin. Biochem..

[138] Antonopoulou S., Fragopoulou E., Karantonis H.C., Mitsou E., Sitara M., Rementzis J., Mourelatos A., Ginis A., Phenekos C. (2006). Effect of Traditional Greek Mediterranean Meals on Platelet Aggregation in Normal Subjects and in Patients with Type 2 Diabetes Mellitus. J. Med. Food.

[139] Karantonis H.C., Fragopoulou E., Antonopoulou S., Rementzis J., Phenekos C., Demopoulos C.A. (2006). Effect of Fast-Food Mediterranean-Type Diet on Type 2 Diabetics and Healthy Human Subjects’ Platelet Aggregation. Diabetes Res. Clin. Pract..

[140] Antonopoulou S., Detopoulou M., Petsini F., Choleva M., Ntzouvani A., Fragopoulou E., Kontogianni M., Georgoulis M., Dimitroglou A., Barkas D. (2025). Consumption of a Mediterranean Lean Fish Enriched with Platelet-Activating Factor Inhibitors Extracted from Olive Pomace Favorably Modulates Hemostasis and Thrombosis in Healthy Adults with Overweight. Food Funct..

[141] Antonopoulou S., Detopoulou M., Fragopoulou E., Nomikos T., Mikellidi A., Yannakoulia M., Kyriacou A., Mitsou E., Panagiotakos D., Anastasiou C. (2022). Consumption of Yogurt Enriched with Polar Lipids from Olive Oil By-Products Reduces Platelet Sensitivity against Platelet Activating Factor and Inflammatory Indices: A Randomized, Double-Blind Clinical Trial. Hum. Nutr. Metab..

[142] Borsoi F.T., Neri-Numa I.A., De Oliveira W.Q., De Araújo F.F., Pastore G.M. (2023). Dietary Polyphenols and Their Relationship to the Modulation of Non-Communicable Chronic Diseases and Epigenetic Mechanisms: A Mini-Review. Food Chem. Mol. Sci..

[143] Hosseini-Esfahani F., Koochakpoor G., Daneshpour M., Sedaghati-khayat B., Mirmiran P., Azizi F. (2017). Mediterranean Dietary Pattern Adherence Modify the Association between FTO Genetic Variations and Obesity Phenotypes. Nutrients.

[144] Aoun C., Hajj A., Hajj F., Papazian T., Rabbaa Khabbaz L. (2022). The Interaction between Genetic Polymorphisms in FTO, MC4R and MTHFR Genes and Adherence to the Mediterranean Diet in Relation to Obesity. Gene.

[145] Di Renzo L., Cioccoloni G., Falco S., Abenavoli L., Moia A., Sinibaldi Salimei P., De Lorenzo A. (2018). Influence of FTO Rs9939609 and Mediterranean Diet on Body Composition and Weight Loss: A Randomized Clinical Trial. J. Transl. Med..

[146] Xiang L., Wu H., Pan A., Patel B., Xiang G., Qi L., Kaplan R.C., Hu F., Wylie-Rosett J., Qi Q. (2016). FTO Genotype and Weight Loss in Diet and Lifestyle Interventions: A Systematic Review and Meta-Analysis. Am. J. Clin. Nutr..

[147] Razquin C., Martinez J.A., Martinez-Gonzalez M.A., Bes-Rastrollo M., Fernández-Crehuet J., Marti A. (2010). A 3-Year Intervention with a Mediterranean Diet Modified the Association between the Rs9939609 Gene Variant in FTO and Body Weight Changes. Int. J. Obes..

[148] Dedoussis G.V., Panagiotakos D.B., Chrysohoou C., Pitsavos C., Zampelas A., Choumerianou D., Stefanadis C. (2004). Effect of Interaction between Adherence to a Mediterranean Diet and the Methylenetetrahydrofolate Reductase 677C→T Mutation on Homocysteine Concentrations in Healthy Adults: The ATTICA Study. Am. J. Clin. Nutr..

[149] Alcala-Diaz J.F., Camargo A., Vals-Delgado C., Leon-Acuña A., Garcia-Fernandez H., Arenas-de Larriva A.P., Perez-Cardelo M., Mora-Ortiz M., Perez-Martinez P., Delgado-Lista J. (2025). MiRNAs as Biomarkers of Nutritional Therapy to Achieve T2DM Remission in Patients with Coronary Heart Disease: From the CORDIOPREV Study. Nutr. Diabetes.

[150] Carpi S., Scoditti E., Massaro M., Polini B., Manera C., Digiacomo M., Esposito Salsano J., Poli G., Tuccinardi T., Doccini S. (2019). The Extra-Virgin Olive Oil Polyphenols Oleocanthal and Oleacein Counteract Inflammation-Related Gene and miRNA Expression in Adipocytes by Attenuating NF-κB Activation. Nutrients.

[151] Hernando-Redondo J., Hernáez Á., Sanllorente A., Pintó X., Estruch R., Salas-Salvadó J., Corella D., Arós F., Martínez-González M.Á., Subirana I. (2025). Mediterranean Diet Modulates Gene Expression of Cholesterol Efflux Receptors in High-Risk Cardiovascular Patients. Mol. Nutr. Food Res..

[152] Gkouskou K., Lazou E., Skoufas E., Eliopoulos A.G. (2021). Genetically Guided Mediterranean Diet for the Personalized Nutritional Management of Type 2 Diabetes Mellitus. Nutrients.

[153] Li J., Guasch-Ferré M., Chung W., Ruiz-Canela M., Toledo E., Corella D., Bhupathiraju S.N., Tobias D.K., Tabung F.K., Hu J. (2020). The Mediterranean Diet, Plasma Metabolome, and Cardiovascular Disease Risk. Eur. Heart J..

[154] Bondia-Pons I., Martinez J.A., de la Iglesia R., Lopez-Legarrea P., Poutanen K., Hanhineva K., Zulet M.d.l.Á. (2015). Effects of Short- and Long-Term Mediterranean-Based Dietary Treatment on Plasma LC-QTOF/MS Metabolic Profiling of Subjects with Metabolic Syndrome Features: The Metabolic Syndrome Reduction in Navarra (RESMENA) Randomized Controlled Trial. Mol. Nutr. Food Res..

[155] Barceló F., Perona J.S., Prades J., Funari S.S., Gomez-Gracia E., Conde M., Estruch R., Ruiz-Gutiérrez V. (2009). Mediterranean-Style Diet Effect on the Structural Properties of the Erythrocyte Cell Membrane of Hypertensive Patients: The Prevencion Con Dieta Mediterranea Study. Hypertension.

[156] Van Meer G., Voelker D.R., Feigenson G.W. (2008). Membrane Lipids: Where They Are and How They Behave. Nat. Rev. Mol. Cell Biol..

[157] Sawyer D.B., Andersen O.S. (1989). Platelet-Activating Factor Is a General Membrane Perturbant. Biochim. Et. Biophys. Acta (BBA) Biomembr..

[158] Flasiński M., Broniatowski M., Wydro P., Hąc-Wydro K., Dynarowicz-Łątka P. (2012). Behavior of Platelet Activating Factor in Membrane-Mimicking Environment. Langmuir Monolayer Study Complemented with Grazing Incidence X-Ray Diffraction and Brewster Angle Microscopy. J. Phys. Chem. B.

[159] Travers J.B., Gomez-Cambronero J., Frohman M.A. (2019). Platelet-Activating Factor as an Effector for Environmental Stressors. Lipid Signaling in Human Diseases.

[160] Pollet H., Conrard L., Cloos A.-S., Tyteca D. (2018). Plasma Membrane Lipid Domains as Platforms for Vesicle Biogenesis and Shedding?. Biomolecules.

[161] Lang P.A., Kempe D.S., Tanneur V., Eisele K., Klarl B.A., Myssina S., Jendrossek V., Ishii S., Shimizu T., Waidmann M. (2005). Stimulation of Erythrocyte Ceramide Formation by Platelet-Activating Factor. J. Cell Sci..

[162] Göggel R., Winoto-Morbach S., Vielhaber G., Imai Y., Lindner K., Brade L., Brade H., Ehlers S., Slutsky A.S., Schütze S. (2004). PAF-Mediated Pulmonary Edema: A New Role for Acid Sphingomyelinase and Ceramide. Nat. Med..

[163] Fragopoulou E., Detopoulou P., Alepoudea E., Nomikos T., Kalogeropoulos N., Antonopoulou S. (2021). Associations between Red Blood Cells Fatty Acids, Desaturases Indices and Metabolism of Platelet Activating Factor in Healthy Volunteers. Prostaglandins Leukot. Essent. Fat. Acids.

[164] Detopoulou P., Fragopoulou E., Nomikos T., Antonopoulou S. (2023). Associations of Phase Angle with Platelet-Activating Factor Metabolism and Related Dietary Factors in Healthy Volunteers. Front. Nutr..

[165] Stephens R.W., Arhire L., Covasa M. (2018). Gut Microbiota: From Microorganisms to Metabolic Organ Influencing Obesity. Obesity.

[166] Jonsson A.L., Bäckhed F. (2017). Role of Gut Microbiota in Atherosclerosis. Nat. Rev. Cardiol..

[167] Nemet I., Saha P.P., Gupta N., Zhu W., Romano K.A., Skye S.M., Cajka T., Mohan M.L., Li L., Wu Y. (2020). A Cardiovascular Disease-Linked Gut Microbial Metabolite Acts via Adrenergic Receptors. Cell.

[168] Zhu Y., Li Q., Jiang H. (2020). Gut Microbiota in Atherosclerosis: Focus on Trimethylamine N-oxide. APMIS.

[169] Trichopoulou A., Martínez-González M.A., Tong T.Y., Forouhi N.G., Khandelwal S., Prabhakaran D., Mozaffarian D., de Lorgeril M. (2014). Definitions and Potential Health Benefits of the Mediterranean Diet: Views from Experts around the World. BMC Med..

[170] Rizzoli R., Biver E. (2024). Role of Fermented Dairy Products in the Health Benefits of a Mediterranean Diet. Aging Clin. Exp. Res..

[171] Millman J.F., Okamoto S., Teruya T., Uema T., Ikematsu S., Shimabukuro M., Masuzaki H. (2021). Extra-Virgin Olive Oil and the Gut-Brain Axis: Influence on Gut Microbiota, Mucosal Immunity, and Cardiometabolic and Cognitive Health. Nutr. Rev..

[172] Merra G., Noce A., Marrone G., Cintoni M., Tarsitano M.G., Capacci A., De Lorenzo A. (2020). Influence of Mediterranean Diet on Human Gut Microbiota. Nutrients.

[173] Khavandegar A., Heidarzadeh A., Angoorani P., Hasani-Ranjbar S., Ejtahed H.-S., Larijani B., Qorbani M. (2024). Adherence to the Mediterranean Diet Can Beneficially Affect the Gut Microbiota Composition: A Systematic Review. BMC Med. Genom..

[174] Scaglione S., Di Chiara T., Daidone M., Tuttolomondo A. (2025). Effects of the Mediterranean Diet on the Components of Metabolic Syndrome Concerning the Cardiometabolic Risk. Nutrients.

[175] Mitsou E.K., Kakali A., Antonopoulou S., Mountzouris K.C., Yannakoulia M., Panagiotakos D.B., Kyriacou A. (2017). Adherence to the Mediterranean Diet Is Associated with the Gut Microbiota Pattern and Gastrointestinal Characteristics in an Adult Population. Br. J. Nutr..

[176] Chen Z., Liang N., Zhang H., Li H., Guo J., Zhang Y., Chen Y., Wang Y., Shi N. (2024). Resistant Starch and the Gut Microbiome: Exploring Beneficial Interactions and Dietary Impacts. Food Chem. X.

[177] Feng Y., Xu D. (2023). Short-Chain Fatty Acids Are Potential Goalkeepers of Atherosclerosis. Front. Pharmacol..

[178] Parada Venegas D., De la Fuente M.K., Landskron G., González M.J., Quera R., Dijkstra G., Harmsen H.J.M., Faber K.N., Hermoso M.A. (2019). Short Chain Fatty Acids (SCFAs)-Mediated Gut Epithelial and Immune Regulation and Its Relevance for Inflammatory Bowel Diseases. Front. Immunol..

[179] Pérez-Reytor D., Puebla C., Karahanian E., García K. (2021). Use of Short-Chain Fatty Acids for the Recovery of the Intestinal Epithelial Barrier Affected by Bacterial Toxins. Front. Physiol..

[180] Koeth R.A., Wang Z., Levison B.S., Buffa J.A., Org E., Sheehy B.T., Britt E.B., Fu X., Wu Y., Li L. (2013). Intestinal Microbiota Metabolism of L-Carnitine, a Nutrient in Red Meat, Promotes Atherosclerosis. Nat. Med..

[181] Detopoulou P., Panagiotakos D.B., Antonopoulou S., Pitsavos C., Stefanadis C. (2008). Dietary Choline and Betaine Intakes in Relation to Concentrations of Inflammatory Markers in Healthy Adults: The ATTICA Study. Am. J. Clin. Nutr..

[182] Heianza Y., Ma W., Manson J.E., Rexrode K.M., Qi L. (2017). Gut Microbiota Metabolites and Risk of Major Adverse Cardiovascular Disease Events and Death: A Systematic Review and Meta-Analysis of Prospective Studies. J. Am. Heart Assoc..

[183] Tang W.H.W., Wang Z., Levison B.S., Koeth R.A., Britt E.B., Fu X., Wu Y., Hazen S.L. (2013). Intestinal Microbial Metabolism of Phosphatidylcholine and Cardiovascular Risk. N. Engl. J. Med..

[184] Seldin M.M., Meng Y., Qi H., Zhu W., Wang Z., Hazen S.L., Lusis A.J., Shih D.M. (2016). Trimethylamine N-Oxide Promotes Vascular Inflammation Through Signaling of Mitogen-Activated Protein Kinase and Nuclear Factor-κB. J. Am. Heart Assoc..

[185] Zhu W., Gregory J.C., Org E., Buffa J.A., Gupta N., Wang Z., Li L., Fu X., Wu Y., Mehrabian M. (2016). Gut Microbial Metabolite TMAO Enhances Platelet Hyperreactivity and Thrombosis Risk. Cell.

[186] Skye S.M., Zhu W., Romano K.A., Guo C.J., Wang Z., Jia X., Kirsop J., Haag B., Lang J.M., DiDonato J.A. (2018). Microbial Transplantation With Human Gut Commensals Containing CutC Is Sufficient to Transmit Enhanced Platelet Reactivity and Thrombosis Potential. Circ. Res..

[187] Torres E.R., Wilcox J., Tang W.H.W. (2026). Gut—heart Axis: Emerging Therapies Targeting Trimethylamine N-Oxide Production. Gut Microbes.

[188] Ma R., Fu W., Zhang J., Hu X., Yang J., Jiang H. (2021). TMAO: A Potential Mediator of Clopidogrel Resistance. Sci. Rep..

[189] Krishnan S., O’Connor L.E., Wang Y., Gertz E.R., Campbell W.W., Bennett B.J. (2022). Adopting a Mediterranean-Style Eating Pattern with Low, but Not Moderate, Unprocessed, Lean Red Meat Intake Reduces Fasting Serum Trimethylamine N-Oxide (TMAO) in Adults Who Are Overweight or Obese. Br. J. Nutr..

[190] Deniz M.Ş., Baş M. (2025). Short-Term Mediterranean Dietary Intervention Reduces Plasma Trimethylamine-N-Oxide Levels in Healthy Individuals. Nutrients.

[191] Griffin L.E., Djuric Z., Angiletta C.J., Mitchell C.M., Baugh M.E., Davy K.P., Neilson A.P. (2019). A Mediterranean Diet Does Not Alter Plasma Trimethylamine N-Oxide Concentrations in Healthy Adults at Risk for Colon Cancer. Food Funct..

[192] Costabile G., Vetrani C., Bozzetto L., Giacco R., Bresciani L., Del Rio D., Vitale M., Della Pepa G., Brighenti F., Riccardi G. (2021). Plasma TMAO Increase after Healthy Diets: Results from 2 Randomized Controlled Trials with Dietary Fish, Polyphenols, and Whole-Grain Cereals. Am. J. Clin. Nutr..

[193] Guasch-Ferré M., Hu F.B., Ruiz-Canela M., Bulló M., Toledo E., Wang D.D., Corella D., Gómez-Gracia E., Fiol M., Estruch R. (2017). Plasma Metabolites From Choline Pathway and Risk of Cardiovascular Disease in the PREDIMED (Prevention With Mediterranean Diet) Study. J. Am. Heart Assoc..

[194] Gao P., Rinott E., Dong D., Mei Z., Wang F., Liu Y., Kamer O., Yaskolka Meir A., Tuohy K.M., Blüher M. (2024). Gut Microbial Metabolism of Bile Acids Modifies the Effect of Mediterranean Diet Interventions on Cardiometabolic Risk in a Randomized Controlled Trial. Gut Microbes.

[195] Chiang J.Y., Ferrell J.M. (2020). Bile Acid Receptors FXR and TGR5 Signaling in Fatty Liver Diseases and Therapy. Am. J. Physiol. Gastrointest. Liver Physiol..

[196] Baars A., Oosting A., Knol J., Garssen J., Bergenhenegouwen J.V., Baars A., Oosting A., Knol J., Garssen J., Bergenhenegouwen J.V. (2015). The Gut Microbiota as a Therapeutic Target in IBD and Metabolic Disease: A Role for the Bile Acid Receptors FXR and TGR5. Microorganisms.

[197] Seo S.-K., Kwon B. (2023). Immune Regulation through Tryptophan Metabolism. Exp. Mol. Med..

[198] Mao Y., Kong C., Zang T., You L., Wang L.-S., Shen L., Ge J.-B. (2024). Impact of the Gut Microbiome on Atherosclerosis. mLife.

[199] Cani P.D., Amar J., Iglesias M.A., Poggi M., Knauf C., Bastelica D., Neyrinck A.M., Fava F., Tuohy K.M., Chabo C. (2007). Metabolic Endotoxemia Initiates Obesity and Insulin Resistance. Diabetes.

[200] Violi F., Cammisotto V., Bartimoccia S., Pignatelli P., Carnevale R., Nocella C. (2023). Gut-Derived Low-Grade Endotoxaemia, Atherothrombosis and Cardiovascular Disease. Nat. Rev. Cardiol..

[201] Camussi G., Mariano F., Biancone L., De Martino A., Bussolati B., Montrucchio G., Tobias P.S. (1995). Lipopolysaccharide Binding Protein and CD14 Modulate the Synthesis of Platelet-Activating Factor by Human Monocytes and Mesangial and Endothelial Cells Stimulated with Lipopolysaccharide. J. Immunol..

[202] Liu J., Bátkai S., Pacher P., Harvey-White J., Wagner J.A., Cravatt B.F., Gao B., Kunos G. (2003). Lipopolysaccharide Induces Anandamide Synthesis in Macrophages via CD14/MAPK/Phosphoinositide 3-Kinase/NF-κB Independently of Platelet-Activating Factor. J. Biol. Chem..

[203] Frostegård J., Huang Y.H., Rönnelid J., Schäfer-Elinder L. (1997). Platelet-Activating Factor and Oxidized LDL Induce Immune Activation by a Common Mechanism. Arterioscler. Thromb. Vasc. Biol..

[204] Harishkumar R., Hans S., Stanton J.E., Grabrucker A.M., Lordan R., Zabetakis I. (2022). Targeting the Platelet-Activating Factor Receptor (PAF-R): Antithrombotic and Anti-Atherosclerotic Nutrients. Nutrients.

[205] Antonopoulou S., Mitsou E.K., Kyriacou A., Fragopoulou E., Detopoulou M. (2024). Does Yogurt Enriched with Platelet-Activating Factor Inhibitors from Olive Oil By-Products Affect Gut Microbiota and Faecal Metabolites in Healthy Overweight Subjects? (A Randomized, Parallel, Three Arm Trial.). Front. Biosci..

[206] Wilmanski T., Kornilov S.A., Diener C., Conomos M., Lovejoy J.C., Sebastiani P., Orwoll E.S., Hood L., Price N.D., Rappaport N. (2022). Heterogeneity in Statin Responses Explained by Variation in the Human Gut Microbiome. Med.

[207] Hoffmann Sardá F.A., Giuntini E.B., Oliveira A., Souza G.S., Prado S.B.R., Taddei C.R., Tadini C.C., Bittinger K., Bushman F.D., Menezes E.W. (2025). Baseline Intestinal Microbiota Composition Influences Response to a Real-World Dietary Fiber Intervention. npj Biofilms Microbiomes.

[208] Jimenez-Torres J., Alcalá-Diaz J.F., Torres-Peña J.D., Gutierrez-Mariscal F.M., Leon-Acuña A., Gómez-Luna P., Fernández-Gandara C., Quintana-Navarro G.M., Fernandez-Garcia J.C., Perez-Martinez P. (2021). Mediterranean Diet Reduces Atherosclerosis Progression in Coronary Heart Disease: An Analysis of the CORDIOPREV Randomized Controlled Trial. Stroke.

[209] Karantonis H.C., Antonopoulou S., Perrea D.N., Sokolis D.P., Theocharis S.E., Kavantzas N., Iliopoulos D.G., Demopoulos C.A. (2006). In Vivo Antiatherogenic Properties of Olive Oil and Its Constituent Lipid Classes in Hyperlipidemic Rabbits. Nutr. Metab. Cardiovasc. Dis..

[210] Schwingshackl L., Hoffmann G. (2014). Monounsaturated Fatty Acids, Olive Oil and Health Status: A Systematic Review and Meta-Analysis of Cohort Studies. Lipids Health Dis..

[211] Antonopoulou S. (2025). Revisiting the Role of Platelet-Activating Factor in COVID-19-Induced Cardiovascular Complications. Front. Biosci..

[212] Nasopoulou C., Tsoupras A.B., Karantonis H.C., Demopoulos C.A., Zabetakis I. (2011). Fish Polar Lipids Retard Atherosclerosis in Rabbits by Down-Regulating PAF Biosynthesis and up-Regulating PAF Catabolism. Lipids Health Dis..

[213] Wohlgemuth L., Knapp C.L., Vidoni L., Hug S., Müller P., Mohamed A.O.K., Dietz A., Stratmann A.E.P., Stukan L., Höpfer L.M. (2025). Platelet-Activating Factor Promotes Neutrophil Activation and Platelet–Neutrophil Complex Formation. Scand. J. Immunol..

[214] Karantonis H.C., Antonopoulou S., Demopoulos C.A. (2002). Antithrombotic Lipid Minor Constituents from Vegetable Oils. Comparison between Olive Oils and Others. J. Agric. Food Chem..

[215] Stamatakis G., Tsantila N., Samiotaki M., Panayotou G.N., Dimopoulos A.C., Halvadakis C.P., Demopoulos C.A. (2009). Detection and Isolation of Antiatherogenic and Antioxidant Substances Present in Olive Mill Wastes by a Novel Filtration System. J. Agric. Food Chem..

[216] Panayiotou A., Samartzis D., Nomikos T., Fragopoulou E., Karantonis H.C., Demopoulos C.A., Zabetakis I. (2000). Lipid Fractions with Aggregatory and Antiaggregatory Activity toward Platelets in Fresh and Fried Cod (*Gadus morhua*): Correlation with Platelet-Activating Factor and Atherogenesis. J. Agric. Food Chem..

[217] Rementzis J., Antonopoulou S., Argyropoulos D., Demopoulos C.A. (1996). Biologically Active Lipids from S. Scombrus. Platelet-Activating Factor and Related Lipid Mediators 2.

[218] Tsoupras A., Lordan R., Shiels K., Saha S.K., Nasopoulou C., Zabetakis I. (2019). In Vitro Antithrombotic Properties of Salmon (*Salmo salar*) Phospholipids in a Novel Food-Grade Extract. Mar. Drugs.

[219] Koussissis S., Semidalas C., Hadzistavrou E., Kalyvas V., Antonopoulou S., Demopoulos C. (1994). Paf antagonists in foods: Isolation and identification of PAF antagonists in honey and wax. Rev. Française Corps Gras.

[220] Lim H., Kubota K., Kobayashi A., Seki T., Ariga T. (1999). Inhibitory Effect of Sulfur-Containing Compounds in *Scorodocarpus borneensis* Becc. on the Aggregation of Rabbit Platelets. Biosci. Biotechnol. Biochem..

[221] Phillips C., Poyser N. (1978). Inhibition of Platelet Aggregation by Onion Extracts. Lancet.

[222] Nasopoulou C., Gogaki V., Panagopoulou E., Demopoulos C., Zabetakis I. (2013). Hen Egg Yolk Lipid Fractions with Antiatherogenic Properties. Anim. Sci. J..

[223] Antonopoulou S., Semidalas C.E., Koussissis S., Demopoulos C.A. (1996). Platelet-Activating Factor (PAF) Antagonists in Foods: A Study of Lipids with PAF or Anti-PAF-like Activity in Cow’s Milk and Yogurt. J. Agric. Food Chem..

[224] Antonopoulou S., Demopoulos C. (1997). On the Mediterranean Diet. Int. News Fats Oils Relat. Mater..

[225] Thomas S.R., Stocker R. (2000). Molecular Action of Vitamin E in Lipoprotein Oxidation: Implications for atherosclerosis. Free Radic. Biol. Med..

[226] Verouti S.N., Tsoupras A.B., Alevizopoulou F., Demopoulos C.A., Iatrou C. (2013). Paricalcitol Effects on Activities and Metabolism of Platelet Activating Factor and on Inflammatory Cytokines in Hemodialysis Patients. Int. J. Artif. Organs.

[227] Fragopoulou E., Demopoulos C., Antonopoulou S. (2009). Lipid Minor Constituents in Wines. A Biochemical Approach in the French Paradox. Int. J. Wine Res..

[228] Fragopoulou E., Antonopoulou S. (2020). The French Paradox Three Decades Later: Role of Inflammation and Thrombosis. Clin. Chim. Acta.

[229] Fragopoulou E., Antonopoulou S., Demopoulos C.A. (2002). Biologically Active Lipids with Antiatherogenic Properties from White Wine and Must. J. Agric. Food Chem..

[230] Fragopoulou E., Antonopoulou S., Nomikos T., Demopoulos C.A. (2003). Structure Elucidation of Phenolic Compounds from Red/White Wine with Antiatherogenic Properties. Biochim. Biophys. Acta.

[231] Xanthopoulou M.N., Asimakopoulos D., Antonopoulou S., Demopoulos C.A., Fragopoulou E. (2014). Effect of Robola and Cabernet Sauvignon Extracts on Platelet Activating Factor Enzymes Activity on U937 Cells. Food Chem..

[232] Fragopoulou E., Nomikos T., Karantonis H.C., Apostolakis C., Pliakis E., Samiotaki M., Panayotou G., Antonopoulou S. (2007). Biological Activity of Acetylated Phenolic Compounds. J. Agric. Food Chem..

[233] Piano M.R., Marcus G.M., Aycock D.M., Buckman J., Hwang C.-L., Larsson S.C., Mukamal K.J., Roerecke M., on behalf the American Heart Association Council on Lifestyle and Cardiometabolic Health; Council on Cardiovascular and Stroke Nursing; Council on Clinical Cardiology; and Stroke Council (2025). Alcohol Use and Cardiovascular Disease: A Scientific Statement From the American Heart Association. Circulation.

[234] Antonopoulou S., Demopoulos C.A., Iatrou C. (1996). Blood Cardiolipin in Haemodialysis Patients. Its Implication in the Biological Action of Platelet-Activating Factor. Int. J. Biochem. Cell Biol..

[235] Woodard D.S., Ostrom K.K., McManus L.M. (1995). Lipid Inhibitors of Platelet-Activating Factor (PAF) in Normal Human Plasma. J. Lipid Mediat. Cell Signal..

[236] Detopoulou P., Fragopoulou E., Nomikos T., Yannakoulia M., Stamatakis G., Panagiotakos D.B., Antonopoulou S. (2015). The Relation of Diet with PAF and Its Metabolic Enzymes in Healthy Volunteers. Eur. J. Nutr..

[237] Petsini F., Detopoulou M., Choleva M., Kostakis I.K., Fragopoulou E., Antonopoulou S. (2024). Exploring the Effect of Resveratrol, Tyrosol, and Their Derivatives on Platelet-Activating Factor Biosynthesis in U937 Cells. Molecules.

[238] Tsantila N., Karantonis H.C., Perrea D.N., Theocharis S.E., Iliopoulos D.G., Antonopoulou S., Demopoulos C.A. (2007). Antithrombotic and Antiatherosclerotic Properties of Olive Oil and Olive Pomace Polar Extracts in Rabbits. Mediat. Inflamm..

[239] Tsantila N., Karantonis H.C., Perrea D.N., Theocharis S.E., Iliopoulos D.G., Iatrou C., Antonopoulou S., Demopoulos C.A. (2010). Atherosclerosis Regression Study in Rabbits upon Olive Pomace Polar Lipid Extract Administration. Nutr. Metab. Cardiovasc. Dis..

[240] Feliste R., Perret B., Braquet P., Chap H. (1989). Protective Effect of BN 52021, a Specific Antagonist of Platelet-Activating Factor (PAF-Acether) against Diet-Induced Cholesteryl Ester Deposition in Rabbit Aorta. Atherosclerosis.

[241] Zhang J., Chen S., Sun P., Liu Y., Jiang J., Guo C., Cheng J., Liu X., Zhang J., Chen C. (2024). Ginkgolides with Anti-PAF Activity from *Ginkgo biloba* L.. Fitoterapia.

[242] Ye W., Wang J., Little P.J., Zou J., Zheng Z., Lu J., Yin Y., Liu H., Zhang D., Liu P. (2024). Anti-Atherosclerotic Effects and Molecular Targets of Ginkgolide B from Ginkgo Biloba. Acta Pharm. Sin. B.

[243] Nasopoulou C., Karantonis H.C., Perrea D.N., Theocharis S.E., Iliopoulos D.G., Demopoulos C.A., Zabetakis I. (2010). In Vivo Anti-Atherogenic Properties of Cultured Gilthead Sea Bream (*Sparus aurata*) Polar Lipid Extracts in Hypercholesterolaemic Rabbits. Food Chem..

[244] Feng Z., Yang X., Zhang L., Ansari I.A., Khan M.S., Han S., Feng Y. (2018). Ginkgolide B Ameliorates Oxidized Low-density Lipoprotein-induced Endothelial Dysfunction via Modulating Lectin-like ox-LDL-receptor-1 and NADPH Oxidase 4 Expression and Inflammatory Cascades. Phytother. Res..

[245] Petsini F., Ntzouvani A., Detopoulou M., Papakonstantinou V.D., Kalogeropoulos N., Fragopoulou E., Nomikos T., Kontogianni M.D., Antonopoulou S. (2022). Consumption of Farmed Fish, Fed with an Olive-Pomace Enriched Diet, and Its Effect on the Inflammatory, Redox, and Platelet-Activating Factor Enzyme Profile of Apparently Healthy Adults: A Double-Blind Randomized Crossover Trial. Foods.

[246] Detopoulou M., Ntzouvani A., Petsini F., Gavriil L., Fragopoulou E., Antonopoulou S. (2021). Consumption of Enriched Yogurt with PAF Inhibitors from Olive Pomace Affects the Major Enzymes of PAF Metabolism: A Randomized, Double Blind, Three Arm Trial. Biomolecules.

[247] Fragopoulou E., Argyrou C., Detopoulou M., Tsitsou S., Seremeti S., Yannakoulia M., Antonopoulou S., Kolovou G., Kalogeropoulos P. (2021). The Effect of Moderate Wine Consumption on Cytokine Secretion by Peripheral Blood Mononuclear Cells: A Randomized Clinical Study in Coronary Heart Disease Patients. Cytokine.

